# Advanced time-resolved absorption spectroscopy with an ultrashort visible/near IR laser and a multi-channel lock-in detector

**DOI:** 10.2183/pjab.97.014

**Published:** 2021-05-11

**Authors:** Takayoshi KOBAYASHI

**Affiliations:** *1 Center for Neuroscience and Biomedical Engineering, The University of Electro-Communications, Chofu, Tokyo, Japan.; *2 Department of Electrophysics, National Chiao Tung University, Hsinchu, Taiwan.

**Keywords:** ultrashort pulse, ultrafast spectroscopy, optical parametric amplifier, multi-channel lock-in amplifier, real-time vibrational spectrum, phase matching

## Abstract

Ultrashort visible-near infrared (NIR) pulse generation and its applications to ultrafast spectroscopy are discussed. Femtosecond pulses of around 800 nm from a Ti:sapphire laser are used as a pump of an optical parametric amplifier (OPA) in a non-collinear configuration to generate ultrashort visible (500–780 nm) pulses and deep-ultraviolet (DUV, 259–282 nm) pulses. The visible-NIR pulses and DUV pulses were compressed to 3.9 fs and 10.4 fs, respectively, and used to elucidate various ultrafast dynamics in condensed matter with a sub-10 fs resolution by pump-probe measurements. We have also developed a 128-channel lock-in amplifier. The combined system of the world-shortest visible pulse from the OPA and the lock-in amplifier with the world-largest channel-number can clarify the sub-10 fs-dynamics in condensed matter. This system clarified structural changes in an excited state, reaction intermediate, and a transition state. This is possible even during molecular vibration and reactions *via* a real-time-resolved vibronic spectrum, which provides molecular structural change information. Also, ultrafast dynamics in exotic materials like carbon nanotubes, topological insulators, and novel solar battery systems have been clarified. Furthermore, the carrier-envelope phase in the ultrashort pulse has been controlled and measured.

## 1 Introduction

Light has been a driving force of important fields in modern physics, such as quantum mechanics and relativity theory, while even further extending to studies in many other fields of physics, allowing photons or light to be one of leading actors in these fields. The interactions of light with matter from various aspects have become major fields in physics. Furthermore, they have expanded to other fields of science. Photoreactions play important roles in both chemistry and biology, *i.e.*, photochemical and photobiological reaction processes. In the former process, photosynthesis is the largest-size chemical reaction on Earth and artificial photosynthesis is one of the hottest urgent topics for realizing a sustainable society. In the latter process, human vision provides more than ∼70% of information from our surroundings. It is essentially important to clarify the “primary” reaction mechanisms in these photochemical and photobiological processes. The reasons for this are described below.

Photoreceptors relevant to the photosynthetic processes can be classified into several types of chlorophyll in chloroplasts (organelle) and various carotenoids. To collect solar energy with a broad spectrum, corresponding to ∼6000 K black-body radiation, they have several types of antenna-like pigments, such as chlorophylls, bacteriochlorophylls, phycobilisomes and carotenoids. The collected photoenergy by the pigments is transferred to the reaction center and drives the chemical reactions of water splitting and carbonic acid assimilation in botanical cells. While in a vision process, a main relevant photoreceptor is rhodopsin, which is classified into two types: dark-and-bright sensing, and three-color sensing. Some invertebrates have pigments with the capability of four-color-sensing, including near-ultraviolet (NUV). The last one is useful for identifying different colors with white (no color for human eyes) flowers to be distinguished by insects. Because of the low illuminance of solar energy on Earth, the absorption cross section of the photoreceptor molecules (both in photosynthesis and vision) is sufficiently large to capture the solar photons. Since the absorption transition is the reverse process to induced emission, a system with a large absorption cross section has a high spontaneous emission rate, given by the A coefficient. The inverse of the Einstein A coefficient corresponding to the spontaneous emission (= fluorescence) lifetime in the visible spectral range is in a nanosecond time scale. Then, it is required for the primary processes of the energy captured by solar light absorption in the excited state to be processed before losing *via* spontaneous emission in a substantially shorter time scale than the fluorescence lifetime. In the same way, an efficient photochemical reaction is required to be faster than the decay, while in the case of photothermal reactions in which the absorbed photon energy is converted to thermal energy, inducing thermal reactions, there is no need for the primary process to take place within the excited state lifetime. Therefore, ultrashort laser pulses in the femtosecond-to-picosecond time regime are required to elucidate the time-resolved mechanism of primary processes in photochemistry and photobiology. But for true photochemical reactions to take place efficiently, its primary process is needed to proceed faster than spontaneous emission.

Even for chemical reactions other than photochemical processes, fast dark (= thermal) chemical reactions cannot be traced by the naked eyes, which have only ∼10 millisecond resolution due to the slow amplification processes in neurophysiology. However, it may become observable by flash photolysis utilizing strobe light with a short duration, as demonstrated by Baron George Porter^1)^ of the Royal Society of London. The time resolution is much better than that of human naked eyes. The resolution time of the flash photolysis method was limited to a microsecond time range due to the duration of the discharge flash lamp at the time. Furthermore, in the experiment, spectroscopic information was used. For detecting the spectrum, a photographic dry plate and a prism monochromator were utilized. The limited time resolution is partly because of the finite time constant of the electric discharging circuit containing high capacitance enabling the high current needed to generate intense flashlight. The flashlight duration is also limited by a disappearance time of ionic species, such as oxygen and nitrogen cations in the discharge plasma. To observe phenomena proceeding faster than the microsecond time scale, it is necessary to use new light sources that can generate shorter light pulses than the flash lamp. A laser (acronym of “light amplification by stimulated emission of radiation” followed after the name of maser from “microwave amplification of stimulated emission of radiation”)^2)^ can be used to generate light with a much higher intensity for many applications including spectroscopy. Even more intense pulses can be generated by the method of “Q-switching”. This is realized by a rapid increase in the cavity Q (quality factor), defined by *Q* = ω_*r*_/Δω = 2π(energy stored/energy dissipated per cycle), with a rotating mirror or by other mechanisms, such as by absorption saturation by which an intense pulse can be obtained. The Q-switched laser enabled the generation of a <10 nanosecond pulse, called a ‘giant pulse’,^3)^ to be used in nanosecond time-resolved absorption spectroscopy. After this development a further time-resolution improvement was realized by the mode-lock method^4)^ generating ultrashort pulses from picosecond to femtosecond duration. Femtosecond laser pulses could be applied for observing the transition states of chemical reactions by Ahmed Zewail^5)^ at California Institute of Technology. This pioneering work was followed by time-resolved studies of various chemical reactions, which has created a research field called “femtochemistry”. This method can also be used for photophysical and photobiological processes, like vision^6)^ and photosynthesis.^7)^ The ultrafast dynamics provides key information related to modern technology especially optoelectronics technologies including the research and development of devices, such as photosensors,^8–10)^ optical memories and image storages,^11–13)^ and optical switches.^14–16)^

## 2 Characteristic properties of ultrashort pulses

When discussing ultrashort pulse lasers, three comments must be mentioned concerning the condition of the short pulse determined by the bandwidth of the source spectrum.

One is about the time-bandwidth relation. The pulse width of an ultrashort pulse is limited by the inverse of the spectral width. Sometimes it is called the Heisenberg uncertainty relation between energy and time. But of course, this is not the case. The Heisenberg relation is between two physical quantities represented by conjugate operators, such as the position and momentum. Since time cannot be an operator, but it is a parameter, the Heisenberg relation, cannot be applied. The uncertainty between the pulse duration and the spectral width is not physical, but mathematical (logic), based on the Fourier-transform (FT) relation between the two. This is called the time-bandwidth product relation given by Δν · Δ*t* ≥ *a*. Here *a* is a specific constant on the order of 1, or smaller, depending on the pulse temporal and spectral shapes. For example, in the case of a Gaussian temporal and spectral pulse, the product is lower limited by *a* = 0.441. In the cases of pulses with shapes of a square (box) function, a squared hyperbolicsecond (sech^2^) function, and a Lorentz function, the FT limited time-bandwidth products, *a*’s, are 0.892, 0.315, and 0.142, respectively.

A physics-based interpretation appears after invoking the relation *E* = *h*ν. From this relation the uncertainty of the energy is given by Δ*E* · Δ*t* ≥ *ha*.

The second comment is about the following question given sometimes to me after my talk on ultrafast spectroscopy, since I am using a very broad spectrum covering the visible (green)-near infrared spectrum region from an ultrashort pulse and a broad-band multi-channel lock-in amplifier (MLA), discussed in the later section of this paper. This can measure the whole spectrum which supports the ultrashort pulse duration. Then, a question is raised, such as “Is the time resolution of the target system limited by Δ*t* = *a*/Δν because of the Heisenberg uncertainty?”. This question can be replied to in the following way. Since the time-resolved difference absorption spectrum obtained is a snapshot at some specific delay time after a pump pulse, the probe can provide sufficient time-resolved broad spectral information. The time-resolved spectrum at a specific “representative” delay time changes in a way satisfying the time-frequency relation. Therefore, it cannot change rapidly from one gate (= probed) delay time to the next. Even in the case that we utilize a high-resolution spectrometer to detect the probe pulse spectrum a spectral change, itself, cannot take place.

As the second point, it must be recognized that the Heisenberg uncertainty principle says that conjugate physical quantities cannot be precisely determined at the same “time”. Therefore, time is not a “quantity” to be determined or measured at some specific time. An electronic transition exists in some atom or molecule, which we refer to as the natural lifetime, τ_nat_. This lifetime is the radiative life excluding radiation-less relaxation, which is given by the inverse of the transition spectral line width in the case the system does not have any inhomogeneity. Based on the measurement of the spectrum by detecting the time-gated collection of components appearing only at a longer delay time after excitation than the natural lifetime, the observed spectral line width is narrower than the natural line width. This is again due to the FT relation. Utilizing this mechanism, sub-natural spectroscopy was developed to obtain a more precise spectral position of a transition in molecules and atoms.

The third point concerns the difference between some specific spectral point and the width of the relevant spectrum. We can clearly state the amount of shift of some vibrational peak or valley or some other characteristic point in the time-resolved electronic (vibronic) spectrum only at the 1-fs delay after the previous delay time. An example of a time-resolved spectrum that we measured will be shown later. In such a case we can specify the wavelength or wavenumber with a limitation of only 1 cm^−1^, determined by the spectrometer. This is more than 1000-times better than the corresponding resolution limited by the FT relation. The reason for the apparent inconsistency is that the spectral “width” calculated by the difference between the two half-intensity points of homogeneously broadened bands cannot be smaller than the Δν = *a*/Δ*t* value. Here, Δ*t* is the lifetime of the species. This can be well understood by considering that the precision of the spectral point positioning is simply determined by the spectral resolution of the spectrometer, which does not have a time resolution. The spectral resolution in this case is irrelevant to the measuring time, which is longer than 1 millisecond even in the shortest case under a general experimental condition, and orders of magnitude longer than the time-resolution discussed here.

## 3 Ti:sapphire laser

For extensive studies of ultrafast processes in various materials, ultrashort-pulse lasers with a broad lasing bandwidth are required. Previously, dye lasers have been utilized as visible laser light sources owing to their material variety and wide tunability of each dye. However, the output power is very low and mechanical instability of the dye “jet” used to avoid any chemical degradation of the dye stuff in solution is so serious that they are not used now except for only limited applications. Solid-state lasers have become much more popular in the field of ultrafast spectroscopy. Among solid-state lasers, the Ti:sapphire laser (Ti: Al_2_O_3_ laser, titanium-sapphire laser, or Ti:sapph-laser) has become the most widely used. The Ti:sapphire laser is a tunable laser which emits red to near-infrared (NIR) light in the range extending from 650 to 1100 nm (nanometer). This laser is widely used in scientific research because of the tunability and capability to generate ultrashort pulses, thanks to the broad gain band width. Lasers based on Ti:sapphire were first constructed in June 1982, by Peter Moulton at the Massachusetts Institute of Technology (MIT) Lincoln Laboratory.^17)^ A titanium-sapphire is the gain medium for lasing. It is a crystal called corundum composed of aluminum oxide (Al_2_O_3_) doped with titanium ions Ti^3+^. Corundum is mechanically hard with a Morse hardness of 9 and has high thermal conductivity. Both properties are well suited to be used as a laser host crystal. The gain medium of the first laser realized is chromium ion (Cr^3+^) doped in the same type of host corundum crystal. A Ti:sapphire laser with an appropriate design can also be used as a continuous wave (CW) laser with extremely narrow linewidths tunable over a wide range of 660–1100 nm. The linewidth is typically <50 kHz and can be at the Hz level *via* an external stable reference. However, nowadays the Ti:sapphire laser is more frequently used for generating ultrashort pulses for high time-resolution which requires a broad-band spectrum supporting the short pulse, as mentioned above. An ultrashort pulse Ti:sapphire laser has become commercially available in the spectral region of NIR corresponding to the gain spectrum of a Ti:sapphire crystal. Commercially available Ti:sapphire lasers typically have an ∼30–100 fs duration and an average power of 0.5 to 2.5 W with a repetition rate of 70–80 MHz.

A Ti:sapphire laser is usually pumped with another laser having an oscillation wavelength between 514 and 532 nm, which is strongly absorbed by the titanium-doped material. The Ti:sapphire oscillator is normally pumped with a continuous-wave (CW) laser beam from an argon-ion gas laser (514.5 nm), a frequency-doubled Nd: YAG, Nd: YLF, or Nd: YVO_4_ laser (527–532 nm), or by optically pumped blue-green diode lasers. Because of convenience, the solid-state laser is nowadays used more frequently, since an Ar laser being used as a pump source can experience problems in its water-cooling system for a meter-long plasma tube. Ti:sapphire lasers operate most efficiently at wavelengths around 800 nm. Mode-locked Ti:sapphire laser oscillators can generate ultrashort pulses with a typical duration of between a few picoseconds and 10 femtoseconds, and in special cases even with a duration of about 5 femtoseconds. Typically, such an oscillator has an average output power of 0.4 to 2.5 watts. The pulse-repetition rate in most cases is from about 70 to 90 MHz, determined by the round-trip time in the laser cavity. Other relatively new solid-state femtosecond lasers, such as Cr^3+^-doped lasers; Cr^3+^: LiCaAlF_6_ (Cr: LiCAF) and Cr^3+^: SrAlF_6_ (Cr: LiSAF) lasers also have a similar gain spectrum in the NIR range.

Cr: LiCAF and Cr: LiSAF lasers can be efficiently pumped by flashlamps, diode lasers, or other lasers. They demonstrate a high laser efficiency (61% with laser pumping, 5% with flashlamp pumping) and a long energy-storage lifetime, which simplifies the pumping requirements, and make the materials suitable for Q-switching and amplifier configurations. The energy storage time is 170 µs in Cr: LiCAF, and is 67 µs in Cr: LiSAF. They possess a large gain bandwidth, allowing the generation and amplification of femtosecond pulses. The peak wavelengths are tunable from 650 to 1180 nm. They have favorable thermomechanical properties, allowing for ease of thermal management and material fabrication. The thermal lensing effect is small due to the weak temperature dependence of the refractive index, leading to good beam quality, even at high power levels. They have very low nonlinear indices (n_2_ = 0.4 × 10^−3^ electrostatic charge units, or esu) and very high damage thresholds (>55 J/cm^2^ at 10 ns), enabling the transmission of undistorted high-intensity pulses through the material.

A further extension of lasing to much longer wavelengths can be made by using transitions between vibrational levels. A carbon dioxide laser is such a case. CO_2_ lasers are the highest-power continuous-wave lasers that are currently available. They are also quite efficient: the ratio of the output power to the pump power can be as large as 20%. The CO_2_ laser produces a beam of infrared light at several wavelengths with the principal bands centering on 9.6 and 10.6 micrometers (µm). Because the excitation energy of the molecular vibrational and rotational mode quantum states is low, the photons emitted due to transitions between these quantum states have a comparatively lower energy, and a longer wavelength than visible and near-infrared light. The 9–12 µm wavelength of a CO_2_ laser is useful because it falls into an important window for atmospheric transmissions (up to 80% atmospheric transmission at the wavelength), and also because many natural and synthetic materials have strong characteristic absorption in this range. The laser wavelength can be tuned by altering the isotopic ratio of the carbon and oxygen atoms comprising the CO_2_ molecules in the discharge tube. The CO_2_ laser can be constructed to have CW powers of between milliwatts (mW) and hundreds of kilowatts (kW). It is also quite easy to actively Q-switch a CO_2_ laser by means of a rotating mirror or an electro-optic switch, giving rise to Q-switched peak powers of up to gigawatts (GW).

To extend the wavelengths of short-pulse laser nonlinear, optical processes such as second-harmonic generation (SHG) and third-harmonic generation (THG) are utilized. Different from an SHG material, which requires a lack of inversion symmetry, THG can be obtained in materials with inversion symmetry even in isotropic media such as gaseous materials. However, the third-order process is very low in such materials. An efficient THG is obtained by a sequential process of the following sum frequency generation of the fundamental and the second-harmonic in a second-order nonlinear crystal without inversion symmetry. There is another way to obtain a short pulse different from the harmonic generation. Ultrashort pulses can be obtained by expanding the bandwidth using the second-order nonlinear optical process, *i.e.*, optical parametric amplification (OPA). In the next section, we discuss femtosecond pulse generation at different wavelengths from the Ti:sapphire laser by a nonlinear optical process *i.e.*, a parametric process of the output from the NIR Ti:sapphire laser.

## 4 Parametric processes and subsidiary effects due to thermal and/or photochemical damage

### 4.1 Parametric interaction.

Ultrafast spectroscopy based on ultrashort pulses exists in the research area located opposite to that of high-(spectral-) resolution spectroscopy. Ultrashort pulses can be obtained from laser systems with a broad gain bandwidth. The laser is a light source utilizing the amplification process by stimulated emission between two states among four states, which enables population inversion of the two states. They are called three-level and four-level lasers; representative lasers are the ruby laser and the Nd: YAG laser, respectively. The relevant two states are usually electronic states in most cases. Lasing at much longer wavelengths can be obtained by stimulated emission between two vibrational levels in a gain medium. A system using vibrational levels was discussed briefly in Section 3 with an example of the carbon dioxide laser. Because of the mechanism, the wavelength is limited by the energy spacing between the two relevant (electronic) states or (vibrational or vibronic) levels. In other words, in the lasing processes the “real states” or “real levels” are involved.

There is another mechanism involving an amplification scheme utilizing “virtual” states or “virtual” levels. These are parametric processes in which “virtual” states (levels) are utilized in the gain processes without invoking “real” states (levels). Thanks to a mitigation of the requirement in parametric processes, there are more chances of finding appropriate materials for amplification in a broad spectral range that can provide the wide tuning and gain bandwidth needed for short-pulse generation.

Because the parametric processes utilize virtual states, which do not have an energy storage mechanism in the media, it requires higher pumping than lasing processes supported by real states. The virtual state has a virtual lifetime determined by the inverse of the detuning energy. It is then at most a few to a few tens of fs, which is several orders of magnitude shorter than the population relaxation time of the electronic excited state.

It is preferred for parametric interactions to take place while utilizing a resonant cavity, in which the interaction time between a photon and the parametric gain material is increased to that of the cavity confinement time. This represents the time that a photon generated in a cavity remains in the cavity, called the “cavity photon lifetime”. Nonlinear optical processes in an optical cavity are assisted by a high field amplitude and/or intensity (= squared absolute value of the field amplitude) due to the cavity confinement, of which the effectiveness is represented by the cavity Q-value. This is given by the ratio between the resonance frequency, ω, and the Full Width at Half Maximum (FWHM), Δω, of the resonance curve, *Q* = ω/Δω = ω*t*_*c*_. Here *t*_*c*_ is the cavity photon lifetime.

Optical parametric (OP) processes include parametric fluorescence (OPF, also called optical parametric generation (OPG)), parametric amplification (OPA), and parametric oscillation (OPO). Among them OPO or OPG is a spontaneous process starting from a vacuum, as in the case of spontaneous fluorescence or spontaneous Raman scattering. In the case of parametric amplification, there are possibilities of utilizing cavity feedback effects to enhance the parametric signal pulse. If the parametric-amplified pulse in the cavity exceeds the threshold, it acts as a parametric oscillation, and if it is below the threshold, but with amplification it is a cavity-assisted optical parametric amplification.

These OP processes have various applications in variety of science and technology, such as quantum information. Spontaneous parametric down conversion is used to generate an entangled pair of photons to be utilized for various applications in quantum information technology. They include quantum imaging, quantum lithography remote measurement, and many others.^18–22)^ In this paper we focus on OPA processes, which are discussed in detail in a later section.

### 4.2 Damage effects.

In laser and parametric amplifier systems, there may be a problem of damage. Such damage processes include the following two types. One is a thermal damage process induced by heat accumulation, which is more serious in the case of a long-pulse laser or a CW laser than pulse systems. The case of short-pulse lasers involves a less-significant problem of thermal damage than long-pulse laser or CW laser, because short pulses can have a high peak power with a moderate average power below the thermal-damage level.

In the case of pulse laser, there is a possibility of damage induced by a high peak power that induces electric breakdown. Short-pulse cases cause another damage problem. This involves two- or multi-step processes. In this case, the first step of photoexcitation creates an excited state that can result in intense absorption to be further excited into higher excited states. Because of multiple succeeding processes, shorter pulses can cause more serious damage because of higher chances of multi-step climbing to higher levels before relaxation. This may result in irreversible photo-induced reactions due to a higher reactivity associated with more electronically delocalized excited states, or due to a higher chance to cross with other excited states being congested due to the higher density of states. In the case of experiencing solid-state laser damage, sometimes we change the laser rod position so as to avoid the intra-cavity beam from travelling through the damaged portion.

The highest care must be taken in order to prevent both types of damage.

## 5 Non-collinear optical parametric amplification (NOPA)

### 5.1 Basics of ultrashort pulse generation by optical parametric amplification (OPA).

Parametric conversion is a general process which can be observed in several physical processes other than optical processes. In classical mechanics, a kid’s swing-pendulum is a good example. If the kid on the swing is stretching his or her legs in synchronization with the swing period, the amplitude increases. If it is anti-synchronization, then the amplitude is reduced. In the former and latter cases, the swing mechanical energy can be periodically transferred to the kid and from the swing to the kid, respectively. Let us imagine a pendulum suspended by a horizontal bar perpendicular to the swinging plane of the pendulum. If the length of string on a horizontal plane is periodically changed by pulling and relaxing with some period different from the pendulum, then some complex oscillation is induced. The period in this case can be analyzed in terms of the mixing of the relevant two periods, their sum and difference frequencies. In examples the period (*T* = 2π(*l*/g)^1/2^) of a pendulum or a kid’s swing is determined by the length (*l*) of the swinging part and the acceleration of gravity (g). The former is a varying “parameter” of the oscillation period of the swing. This is an example of “parametric” coupling in mechanics.

The “optical” version of the parametric interaction processes has drawn the attention of laser scientists during the past several decades because of their capability to generate and amplify the output from the optical parametric (OP) devices with frequencies different from the source laser frequency.

Lasers are excellent light sources because of their high coherence. Depending on the laser systems and conditions the type of coherence is different among them. The coherence is classified into four categories: the optical electric field, ***E***, with four space-time variables, *i.e.*, time, *t*, frequency, ω, space, ***r***, and wave vector, ***k***. Time, *t*, and space, ***r***, span four-dimensional spacetime together. Also, ω and ***k*** span four-dimensional frequency-wavevector Fourier space. A physical quantity, ***A***, can be described in the four-dimensional space-time as ***A*** (***r***, *t*). The FT of ***A***; $FT(A)\overset{\Delta }{=}\tilde{A}$; whose spreads of time, frequency, space, and wave vector are defined as Δ*t*, Δω, Δ***r***, and Δ***k***, respectively. Then, Δ*t* and Δω can have a linear scalar FT relation between them under the “FT limited” condition. FT***r*** and FT***k*** have a three-dimensional vectorial FT relation in the case that the field is spatially homogeneous broadened.

The coherences in the *t* and ***r*** domains are called temporal and space (or spacial) coherence, respectively. In the same way, ω and ***k*** can also have their coherences in the frequency and wave vector domain, respectively.

The coherences of physical quantities are quantitatively described in the following way. The values of ***A***(***r***, *t*) at time *t* and ***A***(***r***, *t* + Δ*t*) at time *t* + Δ*t* are well correlated within the coherence time, Δ*t* < *t*_coh_. The meaning of “correlated” describes the situation that if we know the value of ***A***(***r***, *t*) at time *t* then we can predict the value at *t* + Δ*t*, ***A***(***r***, *t* + Δ*t*). The same can be stated in the case of the relation between ***A***(***r***, *t*) and ***A***(***r*** + Δ***r***, *t*) within the coherence length, Δ***r*** < ***r***_coh_. In principle Δ***r*** and ***r*** wavevector spaces are not necessarily parallel to each other in vector space. In general, ***A***(***r***, *t*) can have a longitudinal, transverse coherence, or both. Also, *A*(***r***, *t*) and $\tilde{A}(\text{k}\text{,}\omega )$ can be three-dimensional quantities. In the case of a coherent optical field with a narrow Δ*t* guarantees a short pulse; a narrow Δω has high monochromaticity, a narrow Δ***r*** has the capability of tight focusing and narrow Δ***k*** can have high directivity. In such a way, a laser can have various coherence properties. Each of them has specific applications, while taking advantage of the properties. Lasers can have more than one such outstanding characteristics at the same time.

The relations between Δ*t* and Δω are given in such a way that the product with the counter parts is limited by the FT relation Δν · Δ*t* = *a*, as mentioned in the previous section, by simply modifying Δω = 2πΔν. In the same way relation between Δ***r*** and Δ***k*** is limited by Δ***r*** · Δ***k*** = *b* with some constant *b* depending on the shapes of the spread functions of ***r*** and ***k***. This manifests the relation between the spreads of Δ***r*** and Δ***k*** in real space and reciprocal space, respectively. The constant *b* is represented with by a 3 × 3 matrix in terms of cartesian coordinates. It can be also with radial coordinates.

The target of this review article is “ultrashort pulse generation for studying ultrafast processes in materials in the visible-near infrared (VIS-NIR) and ultraviolet (UV) regions”, which are useful to characterize the properties of various condensed matter, such as molecules in solutions, organic polymers, and solid-state materials including recently studied exotic materials, such as perovskite and topological insulators as well as super conductors. To study such varieties of materials, it is required to have ultrashort pulse lasers with various spectra and/or widely tunable pulses. This is attained by optical parametric (OP) processes to obtain coherent pulses with a spectrum unavailable from traditional lasers due to the limited energy-level structure in laser materials, which determine the transition energy. The OP processes have grown to wider scopes such as basic scientific research as well as medical and industrial applications.

For such purposes as to extend the spectral range, intense ultrashort pulse sources were developed by employing an optical parametric amplifier (OPA). It was developed by utilizing the three-wave mixing based on second-order nonlinear polarization in a nonlinear crystal. In the process, energy conversion from an intense pump light (ω_p_) to the pair of a weak signal (ω_s_) and an idler ω_i_ = ω_p_ − ω_s_ takes place through the corresponding second-order nonlinear polarizations. The energy of the pump photon couples to the signal and idler photons energies. The former is amplified, and the latter is coherently generated to drive the processes.

In the case of OPA, the pump, signal and idler must satisfy both the energy conservation and the momentum conservation principles. The relations are described in the following equations by simply removing the reduced Planck constant (*ħ* = *h*/(2π)) as follows:

$$\omega_{\text{p}}=\omega_{\text{s}}+\omega_{\text{i}},$$ [1]

$${\text{k}}_{\text{p}}={\text{k}}_{\text{s}}+{\text{k}}_{\text{i}}.$$ [2]

Here, ω_j_ and ***k***_j_ are the angular frequency and the wavevector in a nonlinear crystal with suffixes j = p, s, and i, corresponding to the pump, signal, and idler beam, respectively.

### 5.2 BBO crystal as a gain medium in OPA.

For applying optical parametric amplification in the nonlinear optical crystals, phase-matching (PM) condition among the beams of pump, probe, and idler must be satisfied to obtain efficient amplification. For this purpose, we utilize a nonlinear crystal with birefringence. The birefringence and dichroism are optical properties of materials having different refractive indices and absorptivity, respectively, between two perpendicularly polarized optical beams. Uniaxial and biaxial crystals with one symmetry axis have such properties. Through a birefringent material, optical fields with two perpendicularly polarized beams propagate with different phase velocities (*v*_*ph*_ = *c*/*n* = *k*/ω) due to the different refractive index. A pulsed optical beam propagates through the birefringent material with a group velocity (*v*_*g*_ = *dk*/*d*ω = *c*/*n*_*g*_) different from the phase velocity. Phase-velocity matching (PVM or PM for short) can be satisfied in a birefringent material where the polarizations of the fields and the orientation of the crystal are properly selected^23,24)^ to match their phase velocities. This technique is called “angle tuning”. There is another method to match, *i.e.*, temperature phase matching, which utilizes the different temperature dependence of the refractive indices for the two polarizations. This method can only be used for a nonlinear crystal with a large temperature dependence of the refractive index, *dn*/*dT*. It is also needed to prepare an oven with temperature-stabilized heating equipment with a high transmission window material for both the incident and transmitted beams, which have different wavelengths *via* the OPA process. For efficient amplification of “ultrashort” pulses in the OPA, it is needed for not only phase-matching (PM) condition, but also group-velocity matching (GVM) must be satisfied as discussed later.

Among many nonlinear optical crystals, the BBO (β-BaB_2_O_4_) crystal has an outstanding characteristic, especially in the UV and visible ranges, due to many unique features: wide transparency and phase matching ranges, large second-order nonlinear susceptibility, high damage threshold, and excellent optical homogeneity.^25,26)^ The transparent range extends from 190 nm to 3.5 µm. Therefore, it provides an attractive material for various nonlinear optical applications.

Uniaxial and biaxial crystals have one symmetry axis and two symmetry axes, respectively. The uniaxial crystal is birefringent with two refractive indices of extraordinary and ordinary indices, *n*_e_ and *n*_o_; the former and the latter correspond to perpendicular and parallel polarization propagations, respectively, to the plane formed by the crystal axis and the wave vector. The biaxial crystal is characterized by an indicatrix, which shows that the refractive indices of two perpendicularly polarized light beams have different refractive indices for any propagation direction in the crystal. The shape of the indicatrix is a triaxial ellipsoid. Any vector direction drawn from the origin to the surface of the indicatrix corresponds to the direction for light beam propagation, and the length of the vector is proportional to the refractive index of the crystal, hence proportional to the phase velocity in the direction. Some nonlinear optical crystal has a triaxial symmetry resulting in the complicated beam propagation paths. But in some triaxial system, it can be approximated by a uniaxial symmetric crystal as a quasi-birefringent crystal for simplicity, thanks to a small difference between the two refractive indices out of the three.

In the case of a birefringent or quasi-birefringent nonlinear crystal, there are two types of phase matching; type-I and type-II. In the former and latter, the polarizations of the signal and the idler are, respectively, parallel and perpendicular to each other. In the negative (*n*_e_ < *n*_o_) uniaxial crystal BBO, a type-I (e → o + o) OPA is more suited to ultrashort pulse generation than a type-II (e → o + e) because of easier group-velocity matching (GVM) due to a smaller group velocity mismatch, and a larger effective nonlinear coefficient.^23–26)^ Here (e → o + o) means that that the pump-photon-energy in e-polarization is split into the amplification-energy of the o-polarized signal and the generation energy of the o-polarized idler. Phase matching can be realized by either collinear or non-collinear phase matching according to the beam propagating directions of the pump, signal, and the idler. In the collinear case, all the three beams involved are parallel to each other, while in the non-collinear case the beam propagation directions are not parallel. Collinear phase matching is usually applied to a picosecond or longer pulse laser system, because it is easy to align and for achieving a higher efficiency when the three waves propagate through a longer interacting length. However, in a femtosecond laser system, not only phase matching, which corresponds to (lowest order) phase velocity matching (PVM), but also GVM must be satisfied. The reason is as follows. Because an ultrashort pulse has intrinsically extremely broad spectrum due to the FT relation, a higher-order GVM must also be satisfied to obtain the ultrafast pulse close to the FT limited pulse duration. Therefore, non-collinear phase matching which can provide more parameters to optimize the phase matching, by satisfying the GVM, group velocity dispersion (GVD) compensation, and even higher-order dispersion compensation is preferred.^27–37)^

Parametric processes are discussed above in terms of classical optical physics. Basic quantum electronics and quantum optics views are discussed in the literature.^38–41)^

### 5.3 Optical parametric gain spectrum from a broad-band phase-matched BBO crystal.

The deviation from the phase matching is evaluated from a phase mismatch, Δ*k*, defined by

$$\Delta k≜k_{\text{p}}-k_{\text{s}}\cos\alpha -k_{\text{i}}\cos\beta .$$ [3]

**Figure 1.**
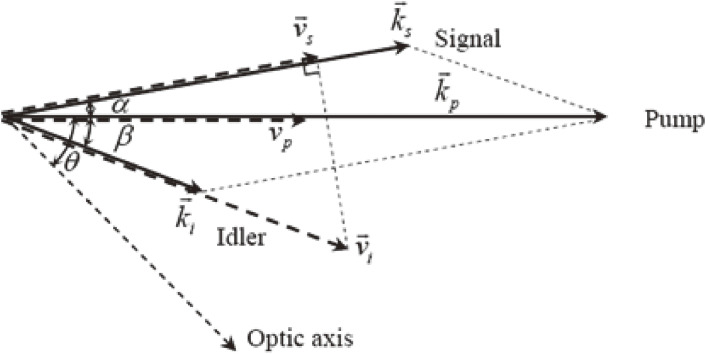
Geometrical configuration of the wave vectors and group velocities in NOPA. Arrows attached to ${\vec{k}}_{j}$ and ${\vec{v}}_{j}$ are the wave vectors (solid lines) and group velocities (thick dashed lines) with the suffixes *j* = *p*, *s* and *i* being the pump, signal and idler, respectively. Angle θ between optic axis and pump wave vector is the phase matching angle. Angles α and β are non-collinear angles between pump and signal and pump and idler, respectively.

Here, α and β are the angles between the wave vectors of the signal *k*_s_ and the pump, *k*_p_, and between the wave vectors of the idler, *k*_i_, and the pump, *k*_p_, respectively, as shown in Fig. 1. In this figure, the phase-matching angle, θ, between the optic axis and the pump beam is also depicted, which defines the pump beam direction with respect to the crystal configuration to obtain the highest efficiency in the OPA process. OPA in this configuration is called non-collinear optical parametric amplification (NOPA). It was developed to obtain shorter pulses by several groups in the world. The history of the development of NOPA is shown in Table 1.

When the phase-matching condition Δ*k* = 0 is satisfied, the parametric gain reaches the maximum because of the longest coherent interaction length. This can be understood in the following way. Within this coherence length, the conversion of the pump photon to the pair of signal and idler photons takes place, while after the coherent interaction length backward process of signal + idler → pump starts to take place resulting in the reduction of the parametric gain efficiency. The unidirectional conversion of pump → signal + idler in the spectral range, where Δ*k* = 0. However, all of *k*_p_, *k*_s_, and *k*_i_ have their intrinsic dispersion relations in a nonlinear crystal, namely the wavelength dependencies of the refractive indices. Since the pump has a relatively narrow spectrum around 800 nm, the dispersion effect can be neglected. If the dependence of Δ*k* on the signal wavelength is small in a certain spectral range, the signal gain maintains high in the range, resulting in an effectively broadband signal amplification. The size of the range is called the parametric gain bandwidth or the parametric bandwidth for short. The broader is the bandwidth, the shorter is pulse width that can be supported according to the FT relation.

Using the plane-wave approximating for the interacting beams and neglecting the pump depletion and finite spectral width of the pump, the well-known parametric gain *G* is given by^41)^

$$\begin{array}{rl} & G=1+(gL{)}^{2}{\left(\frac{\sinh B}{B}\right)}^{2},\\ & B=\sqrt{\Gamma^{2}-{\left(\frac{\Delta k}{2}\right)}^{2}}\cdot L,\\ & \Gamma =2d_{\text{eff}}\sqrt{\frac{\omega_{s}\omega_{i}\Phi }{\varepsilon_{0}n_{s}n_{i}n_{p}c^{3}}}. \end{array}$$ [4]

**Figure 2.**
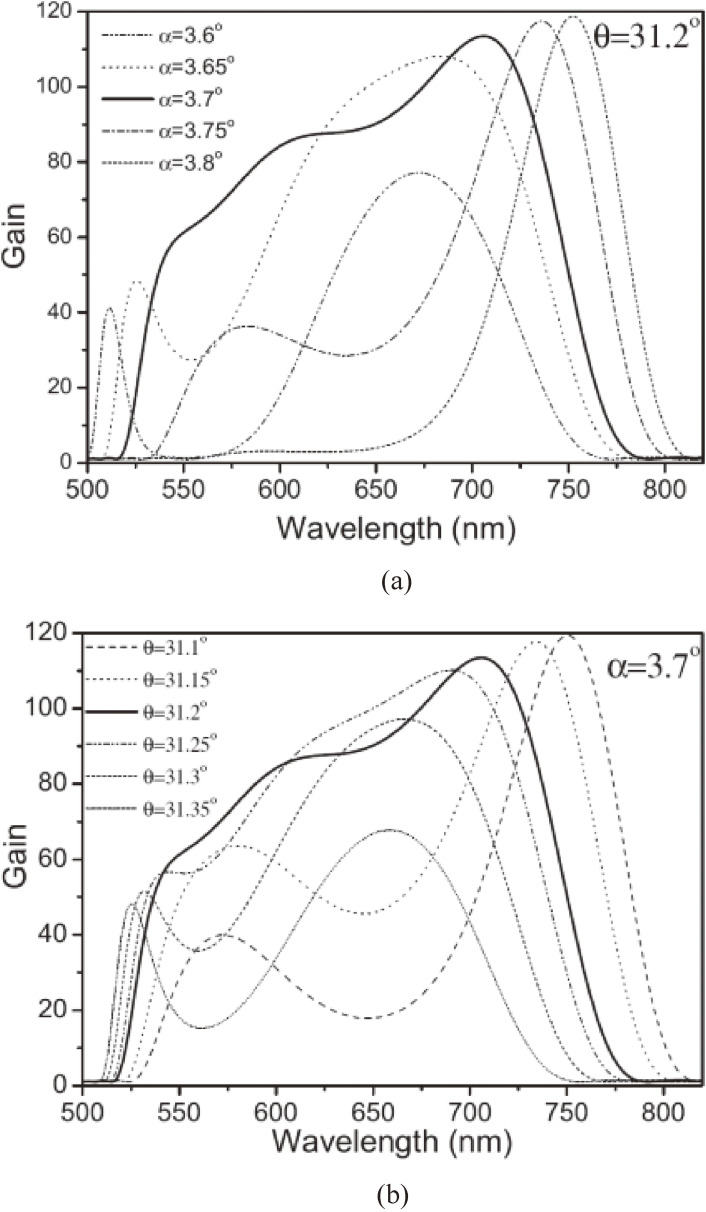
Wavelength dependence of the parametric gain in the type-I non-collinear phase matching condition in a BBO crystal. (a) Varying the non-collinear angle α with a fixed phasing matching angle θ = 31.2°. (b) Varying the phasing matching angle θ with a fixed non-collinear angle α = 3.7°. Thick solid lines in both (a) and (b) are the same and considered to be the best simulation in terms of the broadest gain width and minimum spectral modulation.

Here, *Γ* is the coupling constant among the three beams, *d*_*eff*_ is the effective nonlinear coefficient of the nonlinear optical crystal, ε_0_ is the dielectric constant, *Φ* is the pump intensity and *L* is the nonlinear crystal length; *c* is the speed of light in a vacuum, and *g* is the amplitude gain coefficient of the nonlinear crystal. The gain spectra are calculated for different sets of the nonlinear angle, α, and the phase-matching angle, θ, shown in Fig. 1 using the nonlinear optical parameters of the BBO crystal, *d*_*eff*_ = 1.6 cos θ pm/V, and the pump laser parameter, *Φ* = 50 GW/cm^2^. The wavelength dependence of the gain for several non-collinear angles, α, are shown in Fig. 2(a). The wavelength dependence of the gain at several phase matching-angles, θ, are shown in Fig. 2(b).

Under the present experimental condition with 400 nm for pumping the BBO crystal in type-I OPA, the phase matching angle, θ, and non-collinear angle, α, are set at 31.2° and 3.7°, respectively. The results show that this broad spectrum can support as short as a 5 fs pulse. However, the signal pulse amplified in the BBO crystal has a chirping effect in the pulse, which needs to be compressed by chirp-compensating elements,^42–44)^ such as a prism pair,^43)^ a chirped mirror, and/or a grating pair.^44)^

### 5.4 Pulse front matching configuration in NOPA.

Even after the phase-matching condition is satisfied, there is another factor that restricts the pulse duration to reach the FT limited condition. One of the most dominant effects on the pulse width in the NOPA system is pulse-front tilting, which is an intrinsic effect for the non-collinear interaction of an ultrashort pulse.^27–37,42–46)^

**Figure 3.**
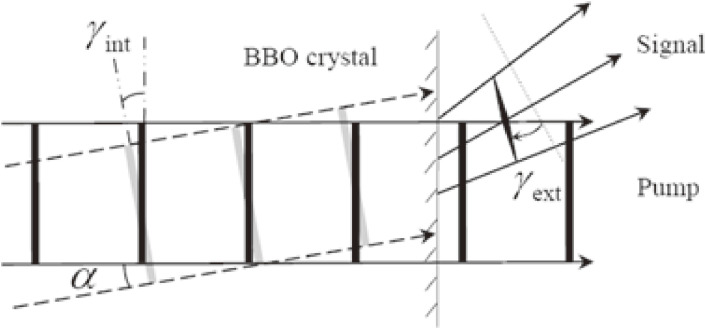
Diagram of the pulse-front tilting between pump and signal in a BBO crystal. The thick solid and gray lines represent the pulse fronts of pump and signal, respectively. The thin line with shadow donates the exit facet of BBO crystal.

Under the non-collinear geometry, the pulse fronts of the pump and signal cannot perfectly overlap with each other, as shown in Fig. 3. The non-collinear amplification causes the tilted gain region volume in the signal beam corresponding to the overlap domain between the pump and signal beams. Between them there is a finite non-collinear angle, α, resulting in the generation of a tilted signal by the same angle, α = γ_int_. In more detail, the angle has some spreading of the beam propagation direction as shown in Fig. 3 because the beam components passing the lower side (in Fig. 3) propagate a longer path length (*l*_lower_) than the higher side (*l*_higher_) in the figure. Therefore, these beamlets have different amounts of group delay, GVD, determined by the group velocity, *v*_group_ (= *c*/*n*_group_ = ∂ω/∂*k* = *c*/(*n* − λ(*dn*/*d*λ))), in terms of the group index, defined by $n_{\text{group}}\;(\overset{\Delta }{=}c/v_{\text{group}})$, while the phase velocity is *v*_phase_ = *c*/*n* = ω/*k*. Due to the wavelength dependence of the group velocity, the group delay ($l_{\text{group}}\overset{\Delta }{=}v_{\text{group}}T_{\text{transit}}$) defined in term of *T*_transit_ also depends on the wavelength. The transit time difference between the upper and lower beamlets in Fig. 3 is defined as *T*_upper_ and *T*_lower_, respectively. Then the transit time difference is given by

$$\begin{array}{rl} \Delta T & =T_{\text{upper}}-T_{\text{lower}}\\ & =\frac{l(n-\lambda \frac{dn}{d\lambda })}{c}-\frac{nl}{c}=-\frac{\lambda }{c}\frac{dn}{d\lambda }l. \end{array}$$ [5]

The relation between the tilted angles inside and outside of the BBO crystal shown in Fig. 3 is

$$\tan\gamma_{\text{int}}=\frac{v_{s}}{c}\tan\gamma_{\text{ext}}.$$ [6]

**Figure 4.**
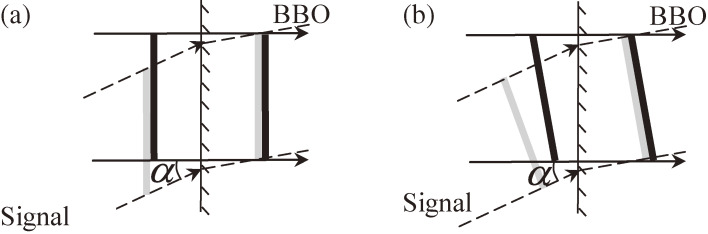
Two schemes for solving the pulse-front mismatching between the pump and signal pulses. (a) Scheme for pre-tilting signal. (b) Scheme for pre-tilting pump.

**Figure 5.**
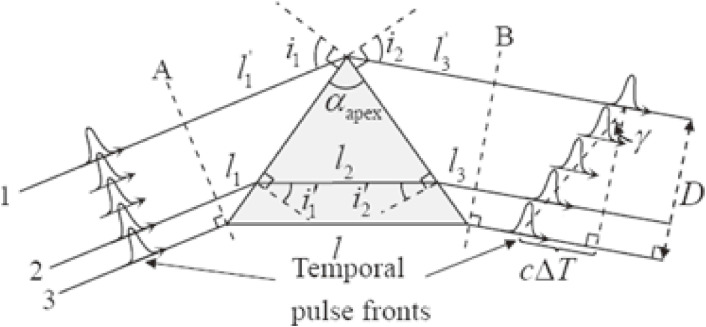
Schematic of the pulse-front tilting during the propagation through a prism. *i*_1_, $i_{1}^{\prime }$, external and internal incident angles; *i*_2_, $i_{2}^{\prime }$ external and internal exit angles. *D* is the beam diameter after the prism. Rays 1 and 3 represent marginal rays propagating through the prism and ray 2 denotes a ray between the two. γ is the pulse front tilting angle after propagating in the prism.

The conversion efficiency decreases with the interaction volume between the pump beam and the signal beam propagating in the nonlinear optical crystal. This volume reduction is substantially due to the above-described pulse-front tilting effect, which also causes the angular dispersion of the exit beam in Fig. 3. There are two ways for the pulse front mismatch shown in Fig. 3 to be reduced, either by a pretilt of the signal pulse front (Fig. 4(a)) or by a pretilt of the pump pulse front (Fig. 4(b)). The second method is preferred because the signal pulse front tilting, exhibiting the finite angle between the wave vector and the Poynting vector after the amplification, needs not to be re-corrected after amplification. Since the angular dispersion also takes place in an analogous way to the light transmission through a prism (Fig. 5) or a grating, we can utilize the angular dispersion in such a simple optical component.

As mentioned above, pre-tilting the pulse fronts can be realized by utilizing simple optical components, such as a prism^43)^ or a grating.^44)^ A prism is preferred because of a much lower Fresnel-reflection loss by prism insertion than a grating loss including reflection loss and a higher-order diffraction loss.

From the geometrical optics formula γ is given by

$$\gamma =\tan^{-1}\left(-\lambda \frac{di_{2}}{d\lambda }\right)=\tan^{-1}\left(-\lambda \frac{di_{2}}{dn}\frac{dn}{d\lambda }\right).$$ [7]

According to a reference^45)^

$$\begin{array}{rl} & \frac{di_{2}}{dn}\\ & =\frac{n\sin\alpha_{\text{apex}}\sqrt{n^{2}-\sin^{2}i_{1}}}{\sqrt{1-[\sin\alpha_{\text{apex}}(n^{2}-\sin^{2}i_{1}{)}^{-\frac{1}{2}}-\sin i_{1}\cos\alpha_{\text{apex}}{]}^{2}}}. \end{array}$$ [8]

Therefore, if the apex angle, α_*apex*_, and light wavelength, λ (of either signal (a) or pump (b) in Fig. 4 depending on the tilting pulse (signal or pump)), are given, expected wave front tilting angle, γ, can be obtained by adjusting the incidence angle *i*_1_ according to Eqs. [7] and [8]. The prism for pulse fronts tilting is usually set before the OPA part, so-called “pre-tilting”.^46)^ This is because after OPA, the intensity is higher and more unstable, and it becomes more difficult to obtain optimum pulse-front tilting condition without, if any, nonlinear optical effects.

### 5.5 Experimental setup of NOPA.

**Figure 6.**
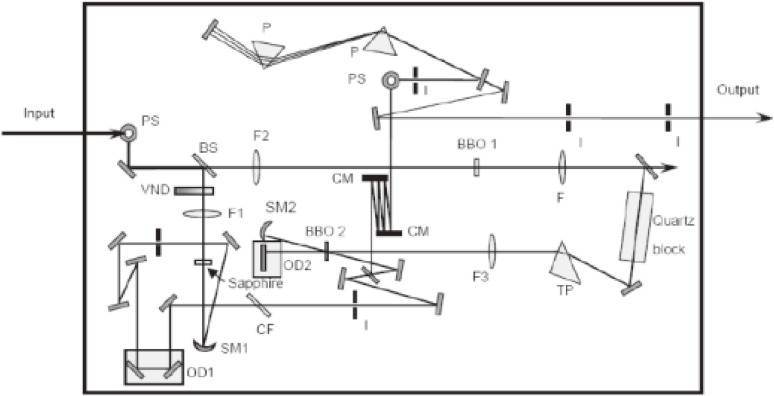
Diagram of NOPA system. PS, Periscope; BS, Beam splitter; VND, Variable neutral-density filter; F's, Focusing lenses (focus length (f) of each lens; F: f = 400 mm; F1: f = 100 mm; F2: f = 400 mm; F3: f = 250 mm); SM's, Spherical mirrors (curvature (r) of each mirror; SM1: r = 120 mm; SM2: r = 100 mm); I, Iris; Sapphire, 2-mm thick sapphire plate to generate a broad-band signal from 450 nm; BBO 1, 0.4 mm BBO crystal to generate second-harmonic light to generate signal beam; BBO 2, 1.0 mm BBO crystal for non-collinear parametric amplification; Quartz block, 10-cm quartz block for pulse stretching; CF, Cut-off filter to remove the wavelength longer than 750 nm; OD1, Adjustable optical delay line for first path OPA; OD2, Adjustable optical delay line for second path OPA; TP, Prism for pulse-front tilting; CM, Chirped mirror; P, Prism to stretch the pulse in pair. The elements without label are simple hard-coated silver plane mirrors.

The experimental setup of NOPA, based on the principle described in the previous subsection, was constructed.^6,30,47)^ The scheme of the system is illustrated in Fig. 6. The pump source of this NOPA system is a commercially supplied regenerative amplifier (Spectra Physics, Spitfire), whose central wavelength, pulse duration, repetition rate, and average output power were 800 nm, 50 fs, 5 kHz, and 750 mW, respectively. The pump beam is introduced from the left in the figure. The input light beam is separated into two parts. One beam transmits through a variable neutral-density filter (VND) to adjust the intensity to an appropriate level. It then propagates through a 2-mm thick sapphire plate (Sapphire) to generate a white-light continuum by a nonlinear optical process due to self-phase modulation (SPM). In general, the continuum generation is mainly due to SPM, but it is not only due to the process, but also due to several other third-order (or even higher) nonlinear processes such as parametric four-wave mixing and stimulated Raman processes. The position of the sapphire plate is carefully adjusted to obtain a stable (in terms of the spectrum, intensity, and spatial transverse modal pattern) continuum and to avoid any air plasma formation at the focal point in case the focus is located outside of the plate. The generated plasma may block the continuum like a plasma shutter. To avoid any air plasma formation, the focal point is usually adjusted after the continuum generation plate, because of the lower intensity than before the plate due to loss induced by reflection and scattering by the plate. A long-cut-off filter that can block wavelengths longer than 750 nm in the pump spectrum is set after the sapphire plate to prevent an intense spectral spike around 800 nm. The second beam propagates through a 0.4 mm-thick BBO crystal (29.2° z-cut, BBO 1) to yield second-harmonic light to pump a NOPA of the type-I (o + o → e) phase-matching condition. To achieve efficient and stable optical parametric amplification, a quartz block is used to stretch the pump pulse width to 200 fs, so as to be comparable to a white-light continuum pulse width maximizing the interaction length. A 45° apex prism is arranged after the quartz block for pre-tilting the pulse front of the pump to be matched with that of the signal in the crystal, as described in Fig. 5 in the previous subsection. Then, the pre-tilted pulse is focused into a 1-mm thick BBO crystal (31.5° z-cut, BBO 2) overlapped spatially with a white-light continuum beam. The optical delay line (OD1) in the white-light continuum beam path is adjusted to have the best temporal overlap. To achieve a higher signal energy, the remaining pump and signal are reflected after the first OPA process and focus once again in the BBO crystal (BBO 2) at a vertically lower (or higher) position while avoiding any overlap with the previous path. The temporal overlapping of the pump and the signal can be regulated by the optical delay line (OD2) in the pump-beam path. The reason for introducing the variable delay line, not in the probe beam, but in the pump beam is to avoid any probe-beam pointing and/or propagation path fluctuation that might affect the quality of the probe intensity, resulting in noise in the spectroscopy signal. Fluctuation of the pump beam affects much less the pump-probe signal since the pump beam diameter is adjusted with a larger focal spot size than that of the probe beam. Because of this configuration, the pass fluctuation in lateral direction of the pump beam is minimized. The effect of any intensity fluctuation of the pump during a femtosecond spectroscopy experiment is reduced by a multi-channel lock-in amplifier to be discussed in the next section.

**Figure 7.**
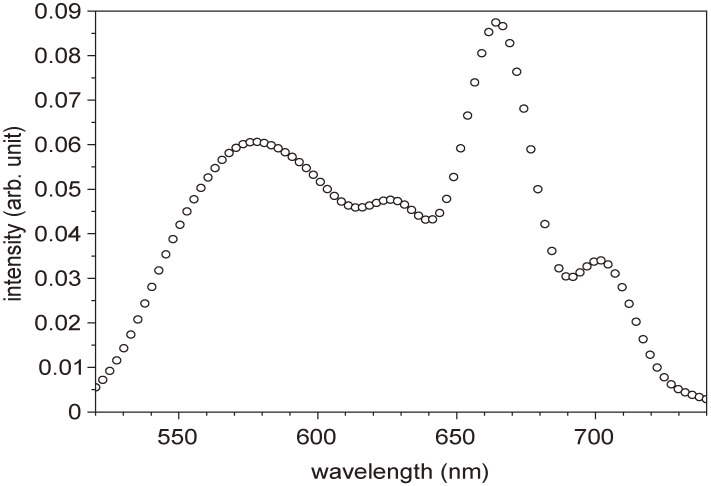
Broad-band visible spectrum generated by NOPA. The output from NOPA is transmitted through a long wavelength (λ > 720 nm) cut-off filter.

The amplified signal just after the BBO crystal (BBO2) is about 0.3 µJ. A pair of ultra-broad-band chirped mirrors (CMs), followed by a prism pair, was designed to compensate for the group delay (GD), group delay dispersion (GDD) and third-order dispersion (TOD) in the NOPA system. The chirped mirrors in the pair are composed of deformable membranes used to adjust the lateral spatial dispersion in the beam cross section. One set of the chirped mirrors was designed to exhibit a GD property with several reflections to compensate the chirp of the NOPA output in the spectral region from 480 to 760 nm in combination with the prism pair, air, and the beam splitter. The other CM set was designed to have GDD and TOD with −45 fs^2^ and 20 fs^3^, respectively, to compensate the dispersion in the spectral region from 500 to 780 nm. These chirped mirror pair and the prism pair compose an optimized compressor. The chirped mirrors have the high reflectivity (R > 99%) with a negligibly small loss, while the prism pair gives the main loss in the present case. The spectrum is adjustable by changing the non-collinear angle, α. The best non-collinear angle, α, was judged experimentally by adjusting the parametric fluorescence ring width to be the thinnest one by visual investigation. Because of the incident angle being deviated from the Brewster angle, there is some inevitable loss, estimated to be about 10%. This is the reason for large loss by the prism pair. The pulse width could be as short as 4.7 fs with a spectrum of 520–730 nm, theoretically. A typical output spectrum of NOPA is shown in Fig. 7. In some experimental cases, the spectrum of NOPA was adjusted to the longer wavelength region extending from 556 to 753 nm to be available to be used for studying some samples of interest with a higher absorbance in the spectral range to be studied. The pulse width in this case is about 7 fs, which is slightly broader than the optimum case.

## 6 Broad-band detection systems for transient absorption spectroscopy

### 6.1 Multi-channel lock-in detection system.

**Figure 8.**
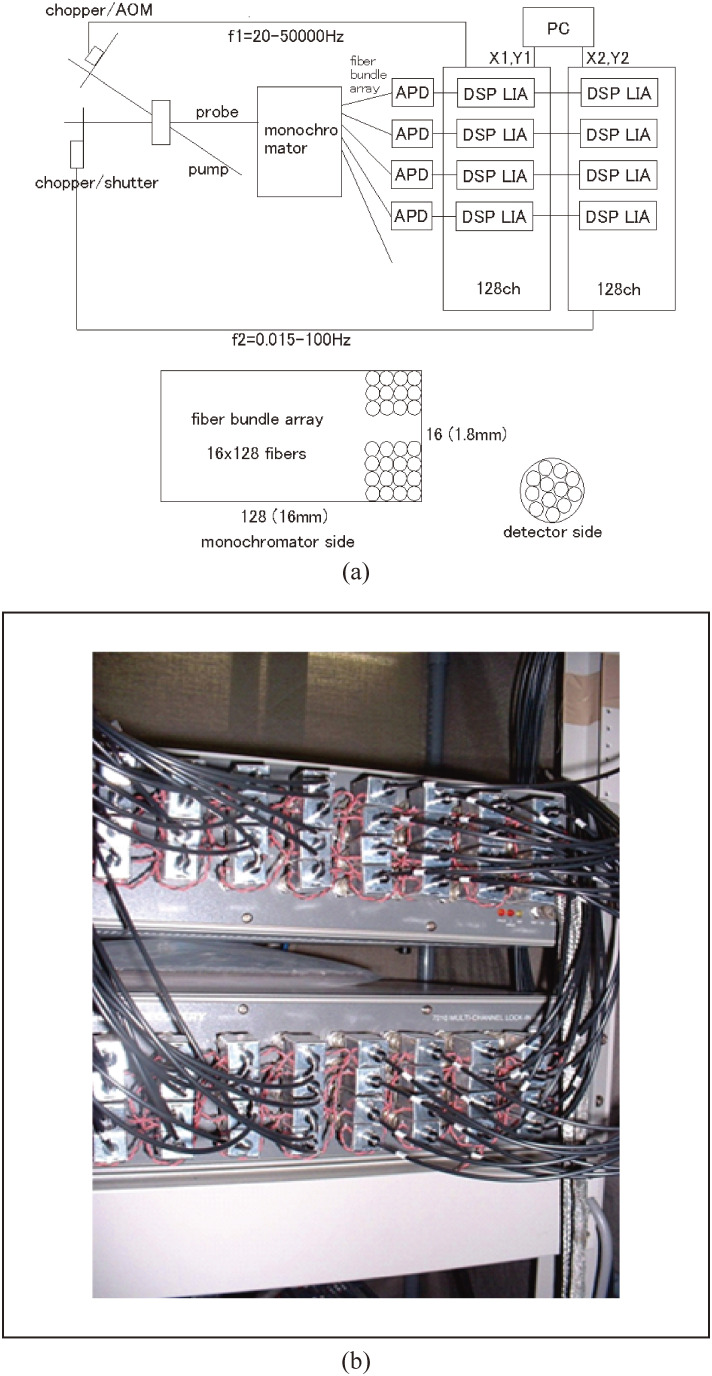
Simplified detection system in the pump-probe experimental system. (a) Probe after being transmitted through amplified sample pump is guided to a monochromator through a fiber bundle array. It is composed of 16 × 128 fibers as shown in the bottom of this figure. The 16 and 128 fiber bundle directions before the monochromator are aligned perpendicular and parallel directions, respectively, of the entrance slit. After the monochromator, the probe light is guided through 16 × 128 fiber bundles aligned at the exit slit position where monochromator provides spectral information of the white probe light. The spectroscopic signal is transferred through 128 channels each of which is coupled to the 16 fibers again. The 16 fiber-sets shown at the bottom of the figure as “detector side” are coupled to 4 sets of APD-DSP LIA. Here DSP LIA is the Digital Signal Processor Lock-In-Amplifier which is composed of 4 sets of 32 channel lock-in amplifiers. The system has the capability of simultaneous multi-wavelength measurements, and it is 128 times faster than single wavelength measurement. (b) External view of the backplanes of the two sets of the four DSP LIA pair-component sets (composed of four pairs of (X1,Y1)-(X2,Y2)).

A quasi-continuum pulse, which is a seed for the NOPA pulse, does not have a very stable spectrum and intensity because of the combined and competing mechanisms of the self-phase modulation, four-wave mixing, stimulated Raman processes, and others. As a result of this instability the NOPA output is also unstable, even though the saturation effect, which is another nonlinear effect, reduces the instability to some extent. To overcome the instability, it is needed for the NOPA to be used for ultrafast time-resolved (TR) spectroscopy; it is a great advantage for a broad-band multi-channel lock-in amplifier (MLA) system to be used to detect the whole TR spectrum at once by integrating over some time constant set for the MLA in TR spectroscopy. The electronics scheme and front panel photo of MLA are shown in Fig. 8(a) and Fig. 8(b), respectively.

Transient absorption spectroscopy (TAS) is one of the powerful tools to study the fast and ultrafast dynamics of relaxation processes in the excited states of condensed matter, molecules and polymer including biological and biomedical systems, and detailed mechanisms of chemical, photochemical and biochemical reactions.

The wavelength dependence of probing can provide much richer information concerning these processes. In the case when we study the probe wavelength dependence of the transient absorption (TA) signal by collecting the signal at each probe wavelength one by one, the measurement condition cannot be maintained among the different probe wavelengths because the femtosecond light source may have intensity and spectral shape instabilities as well as photochemical and/or photophysical damages of the sample may be accumulated in the sample by the irradiation of an intense ultrashort laser pulse. For accurate analysis of the probe wavelength dependency, it is necessary to measure the TA signal at all probe wavelengths simultaneously under the same measurement conditions. This was accomplished by employing a multi-channel detector array discussed below.

The signal of TA spectroscopy includes signal contributions from excited-state absorption (ESA), chemical-reaction intermediate absorption (IA), transient-state absorption (TSA), transient stimulated emission (SE), and ground-state beaching (GSB). These five signal components can be distinguished and separated in principle by studying the probe wavelength dependency of the TA signal. Therefore, we have developed a multi-channel lock-in amplifier (MLA) for phase-sensitive measurements of TA signals at every probe wavelength.^47)^

The MLA was used as a detector of the probe broad-band spectrum generated by self-phase modulation. A probe pulse with a broadband spectrum is focused into a bundled fiber and spectrally dispersed by a polychromator to be coupled into one end of a bundled linear array of 128 fibers, which is then coupled to each of the same number of avalanche photo diodes (APDs) (S5343, Hamamatsu Inc.). The signal detected by each APD is amplified by a corresponding home-made pre-amplifier module, and then the amplified signal is processed by a lock-in amplifier module. Thus, using the 128 pre-amplifiers and 128 lock-in amplifiers, we could collect the TA signal at every probe wavelength simultaneously with a high S/N.^47)^

The combined system of the world-shortest visible pulse laser and the “world-largest channel number” sets of lock-in amplifier can clarify the sub-10 fs dynamics in condensed matter. This system can clarify structural changes in the excited state and the reaction intermediate *via* a real-time-resolved vibronic spectrum. Using the MLA-based TA spectroscopy system, we have elucidated primary excitation and ultrafast relaxation dynamics in various materials.^6,47–88)^ They include exotic materials that are attracting researchers’ interest such as carbon nanotubes,^72,74–77)^ topological insulators,^88)^ and novel solar battery systems.^83)^

**Figure 9.**
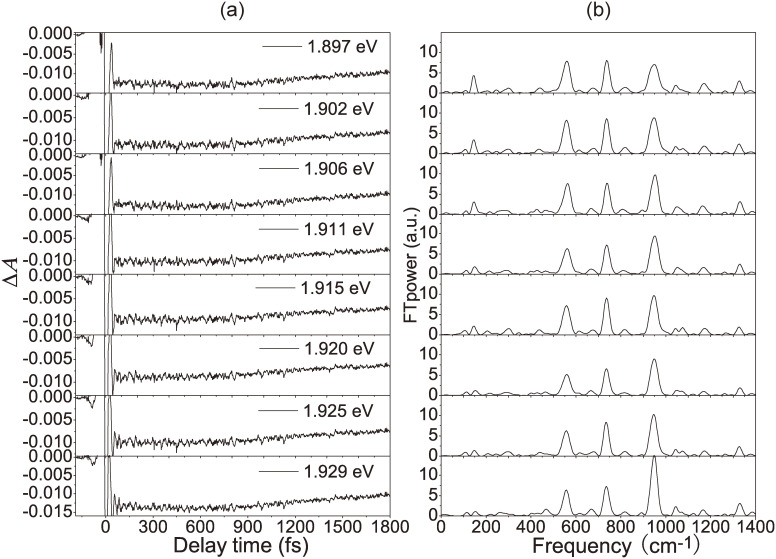
(a) Time-resolved difference absorbance in a cyanine dye (1,1′,3,3,3′,3′-hexamethyl-4,4′,5,5′-dibenzo-2,2′-indotricarbocyanine (HDITC)) in ethanol solution at eight probe photon energies out of 128 channels observed. (b) FT power spectra of the traces in (a).

Out of 128 TR traces of difference absorbance (Δ*A*) obtained at the same experimental time, eight traces are shown as an example in Fig. 9(a) and FT spectrum of the traces in Fig. 9(b). We call this spectrum a “real-time vibrational (amplitude) spectrum”, coupled to the electronic transition *via* vibronic coupling induced by the pump femtosecond pulse. From the gated FT spectra at a specific gate delay time, the molecular structure change at the gate delay can be obtained from the gated FT calculated vibrational spectra.

### 6.2 Optical multi-channel analyzer system.

An Italian group^89)^ developed another type of broad-band detector based on an optical multichannel analyzer with fast electronics to detect energetic probe pulses derived from the NOPA. The sensitivity is sufficient to fill up and “saturate” the detector pixels with a single pulse having ∼25 nJ energy impinging on the detector. This corresponds to about 1 nJ/(10 nm-bandwidth) equivalent to about 66 pJ/pixel in the optical multichannel analyzer. The spectral resolution of the spectrograph is about 2 nm, which is sufficient for ultrafast pump-probe experiments on molecules in the condensed phase at room temperature, since there are no sharp features expected to be observed in the difference transmission spectra. A fast AD conversion card driven by a 2 MHz clock with 16-bit resolution combined with a Peripheral Component Interconnect (PCI) board is installed into the computer used for the experiments. It enables the complete readout of a probe spectrum in 700 µs, thus allowing the recording of single-shot spectra at a 1 kHz-repetition rate. After the data flux has been transferred to the computer and stored in memory, there is still enough time for the software to perform the requested operations such as difference transmittance calculation, averaging, a statistics calculation, and data plotting on a screen. The pump beam is modulated at 500 Hz by a mechanical chopper frequency, which is locked to the laser pulse repetition. This synchronization is achieved by taking the pulse train signal at a 1 kHz generated by the driver of the Pockels cell in the regenerative amplifier and transforming it into a transistor-transistor logic (TTL) 500-Hz square wave with a 50% duty cycle using a binary counter. The pump pulse excites a sample periodically at 2 ms and the probe at 1 ms in between. The time interval is long enough for the sample to be recovered from the photoexcited state.

## 7 Ultrafast pump-probe experiment

### 7.1 Ultrafast pump-probe spectroscopy apparatus.

An extremely simplified block diagram of the pump-probe experimental setup, which is composed of more than 100 components of optics, is shown in Fig. 8(a). For pump-probe experiments, the output from the NOPA is separated into pump and probe beams by a beam splitter at a ratio of 5:1. A frequency divider is synchronized with NOPA and control the chopper at 2.5 kHz, which is half of the pulse repetition rate of NOPA. Both the pump and probe beams are focused on the surface of the sample with about 100 µm and 50 µm, respectively, in diameter by a 12.7 mm (0.5 inch) parabolic mirror (not shown in the figure). The spatial overlap of the pump and probe beams on the sample is carefully adjusted using a pinhole with a 0.1 mm diameter. After the aligning, the pinhole is opened to let the pump beam radius be slightly larger than the probe in a concentric configuration. The transmitted probe beam is coupled into the fiber.

A computer (PC) is prepared for recording the DC (Laser and Transmitted spectra) and AC (Δ*T*(difference transmittance)(*t*, λ)) from the multi-channel lock-in amplifier. The signal is spectrally dispersed using a polychromator (M25-TP, JASCO (shown as “monochromator” in Fig. 8(a))) over 128 channels with a spectral resolution of 1.5 nm; each channel was connected to an avalanche photodiode electronically coupled to a lock-in amplifier locked-in to the 2.5-kHz reference frequency set by an optical chopper that alternatively opens and blocks the pump beam. The polarizations of both the pump and the probe are horizontal in general cases of the solution sample in a glass cell. In case of solid material samples, such as a crystal or doped polymer sample having a perpendicular polarization of the pump and probe, is used to reduce the scattering effects on the sample surface. Perpendicular polarization is obtained by a periscope for the pump. Since stable detection of probe beam is essentially important, the polarization of the pump is controlled in case of, if any, small vibration of the periscope.

The amount of excited and intermediate species proportional to the difference absorbance is Δ*A*(*t*, λ). In the pump-probe experiment we utilize Δ*T*(*t*, λ) instead of Δ*A*(*t*, λ). In the small signal case as always in the present pump-probe experiment, Δ*T*(*t*, λ) ∝ (−Δ*A*) is satisfied and can be used for the amount of ESA, IA, TSA, SE, and GSB. Since the absolute values of the laser intensity at the sample position already have some ambiguity, pump is not necessarily constant in the probe beam cross section on the focal surface due to the intensity distribution across the focal plane. The area of the observation of the nonlinear effects must be clearly described when the sizes of nonlinear effects are described.

**Figure 10.**
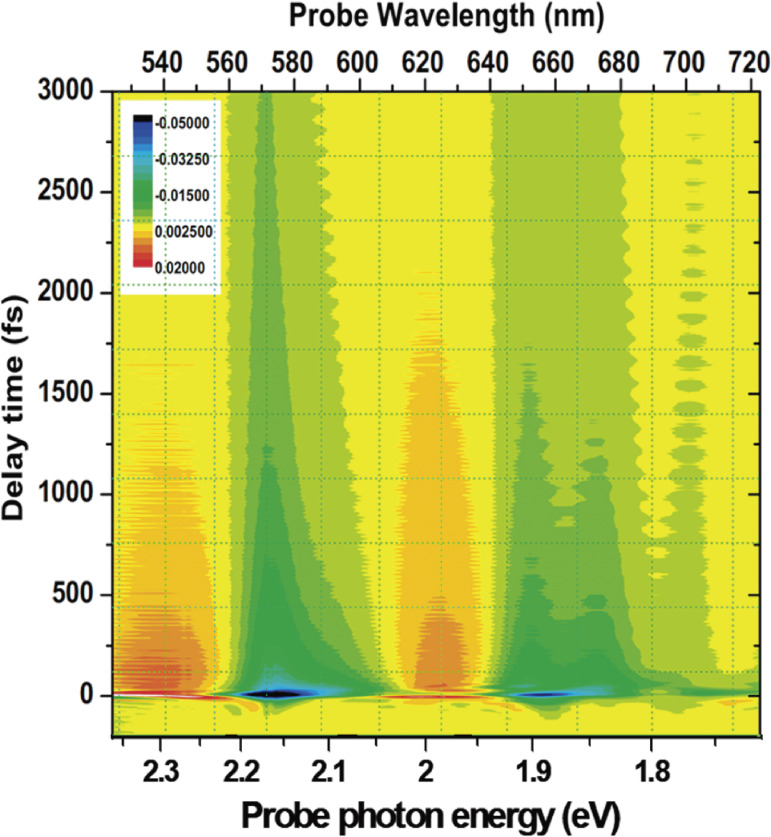
Ultrafast spectral change observed by NOPA with 128 channel lock-in amplifier. The sample is a carbon nanotube excited by intense NOPA and probed with five times weaker intensity NOPA from main NOPA output. The top and bottom abscissas probe wavelength and probe photon energy, respectively. The ordinate is the pump-probe delay time in the range from −200 fs to 3000 fs (= 3 ps). The wavelength (λ) dependent FT spectrum δΔ*A*(ω, λ), calculated from the δΔ*A*(*t*, λ) data, was utilized for the identification to a different ring-diameter carbon nanotube with different ring-breathing mode frequency inversely proportional to the diameter..

Using the ultrashort laser pulse generated by NOPA, pump-probe measurements for various systems have been performed.^6,47–89)^ As an example of the time-resolved spectrum obtained with the NOPA and MLA system, two-dimensionally displayed difference absorption spectrum Δ*A*(*t*, λ) is measured for carbon nanotube as shown in Fig. 10. Cross sectional spectrum of intensity along the line parallel to the horizontal axis of this 2D display corresponds to a time-resolved spectrum at the delay time location of the line. The 2D spectrum shows equidistant (along the delay time direction) stripe-like structure. It is due to the modulated electronic absorbance change Δ*A*(*t*, λ) represented by δΔ*A*(*t*, λ) by molecular vibration. The observed time-dependent absorbance change is thus Δ*A*(*t*, λ) + δΔ*A*(*t*, λ). FT of the modulation provides the information of vibration. Furthermore, the spectrogram obtained by a short-time gated FT provides the information of the molecular structural change which reveals the real-time dynamics of the molecules and polymers being studied. The TR spectroscopy system makes it possible to obtain full Δ*A*(*t*, λ) with a single scanning of probe pulse. This is discussed in the next subsection. Ultrafast dynamics can be time-resolved with the limited resolution to femtosecond in the fs spectroscopy, but even faster processes are of interest; for example, electron-cloud motion, which can be time-resolved by an attosecond pulse to be very briefly discussed in Section 8.^90–93)^ Higher-order nonlinear process, such as higher-harmonics generation (HHG) in deep UV pulse and X-ray pulse generation^90–109)^ is one of the key technologies needed to extend the frequency domain to a higher frequency than that of conventional lasers.

### 7.2 Ultrafast spectroscopy of photophysical, photochemical and photobiological processes.

The duration of a NOPA output pulse can be shorter than the shortest molecular vibration periods of about 10 fs. By combining the world-shortest visible-NIR pulse and lock-in amplifier with 128 channels, discussed in Sections 5 and 6, respectively, detailed information about molecular structural changes in both the ground and excited states as well as reaction intermediates can be clarified *via* “real-time” vibrational (RTV) spectroscopy. Here, “real-time” means the real-time resolved instantaneous vibrational amplitude in the time domain being detected *via* vibration-modulated electronic transition due to ESA, IA, TSA, SE, and GSB. From the instantaneous amplitude, the vibrational phase can also be obtained. Furthermore, by the gated FT (GFT) of the instantaneous vibrational amplitude in the time domain, the vibrational frequency change can be gate-time-resolved to provide the instantaneous molecular structure change.

From RTV spectroscopy, we can observe changes in the vibrational frequency, even during chemical reactions, and then the mechanisms of various photochemical and photobiological reaction can be studied by observing the structural change of molecules, polymers, and other various systems. This is advantageous over conventional “time-resolved” vibrational spectroscopy, such as time-resolved Raman-scattering spectroscopy and time-resolved IR-absorption spectroscopy in which time resolution cannot be better than a few-times longer than the vibrational periods. It is based on the frequency domain measurement and frequency must be defined clearly, requiring information obtained for the least several vibrational periods. The spectrum obtained in Raman and IR spectroscopy is already losing real-time amplitude information by spectrometers, themselves. The real-time vibrational spectrum can analyze even sub-period transient frequency changes due to molecular structural changes or due to the coupling between two or more vibrational modes. RTV spectroscopy obtained by using the gated FT (GFT) analysis molecular structural change during a reaction or during internal-conversion from the Franck-Condon (FC) excited singlet state to the lower states, and then to the lowest excited state, and further to the ground state (S_0_). The gate width can be properly selected and changed at will after pump-probe experimental data are obtained. For example, GFT analysis in RTV spectroscopy of azo dye is described briefly below.

The molecule azobenzene is widely used for blue-color dye material in, for example, “blue jeans” for young people while appreciating the thermal and photochemical stability. We found that the stability mechanism is due to the reversible photoconversion of *trans*-azobenzene to *cis* form resulting not in an irreversible chemical reaction of any kind of photo-degradation or photothermal degradation but in photoenergy dissipation simply to heat.^66)^ There was an argument concerning two candidate mechanisms of the *trans-cis* isomerization pathways from theoretical viewpoints. One is rotation around the N=N bond and the other is the “inversion” like the umbrella motion in an ammonia (NH_3_) molecule, which is a quantum mechanical tunneling process. The real-time vibrational spectroscopy has successfully resolved the vibrational structure “during” isomerization, and found that the reaction takes place with the two coupled modes of rotation and inversion through the lowest-potential barrier passage on the potential “hyper”-surface.^66)^

Various studies can be performed utilizing real-time-resolved vibronic and vibrational spectra in many systems.^47–89)^ They include such systems as photobiological processes in vision and relevant biopolymers,^60,68)^ chlorophyll,^67)^ heme protein,^64)^ RNA bases,^78)^ artificial photosynthetic systems.^83)^ A time-resolved study has been further extended to dynamics in carbon systems, like carbon nanotubes^72,74,77)^ and exotic materials, like topological insulators^89)^ as well as novel solar battery systems strongly desired for the future.^88)^

### 7.3 Transition state spectroscopy.

Ultrafast spectroscopy using the developed ultrashort pulse lasers and multi-channel detection systems, which are described in Sections 5 and 6, respectively, enabled the identifying the intermediate states, and even transition states during ultrafast photochemical and thermochemical reactions. The transition state is considered to be a vibronic wavepacket passing transitionally over the top of a potential barrier.^110–112)^ It is considered that at the top of the potential barrier locally-bound wavepacket is not able to exist, since the potential is convex toward higher energy. However, this is not the case, since if the system is a molecule composed of *N* atoms, then it has 3*N*-6 (nonlinear molecules) or 3*N*-5 (linear molecules) normal coordinates; even if it is along an unbound convex coordinate it can have stable concave potential curves along other coordinates. Therefore, the observation is due a to vibronic wavepacket passing over the saddle point of multi-dimensional potential curves. One example the study concerning the transition spectroscopy of DNA bases to clarify the mechanism of extreme photostability against ultraviolet (UV) light.^78)^ UV-excited DNA molecules have an unusually short lifetime and this is considered to be the reason for them to have become able to survive under intense UV light in the primitive Earth environment before photosynthesis started to generate oxygen, which was converted to ozone. After that ozone became abundant enough for the UV light to be absorbed by atmospheric ozone, resulting in the protection of UV photo-destruction of biological systems. It can be thought that such robust properties of DNA base molecules are due to their ultrashort lifetime, but this was not experimentally verified because such ultrafast spectroscopy in UV region was not available. We developed deep UV (DUV) pulses with a spectral spread of 259–282 nm with 10.4-fs duration, and we could study time- and spectrum-resolved excited states of DNA molecules. We could clarify the ultrashort-lifetime mechanism in terms of potential surfaces of two neighboring lowest excited states experimentally (nπ* and ππ*) and the mechanism is supported by the quantum chemical calculations.^78)^

Our system can be used to study not only the dynamics in the electronic excited states and photochemical reactions but also dark (thermal) reactions. By using the advantage of the ultrabroad spectrum associated with an ultrashort pulse, we can utilize the pulse to excite vibrational excited levels in the electronic ground state *via* a stimulated Raman process. In this process, ultrashort pulses have a sufficiently broad spectral width, supporting two spectral components which work as a pair of pump and Stokes pulses, resulting in the excitation to a vibrational wavepacket composed of two vibrational levels. All of the level pairs with equal or quasi-equal difference energy can be excited forming a vibrational wavepacket. Then, a dark chemical reaction-promoting mode may be excited to start a thermal reaction. Based on this idea, one of the popular ultraviolet reactions, the Claisen rearrangement was studied.^69,73)^ It is important to recognize that this reaction is a coherent-vibrationally excited “thermal reaction”, which is quite different from the conventional incoherent thermal excitation induced chemical reaction triggered by heating. This reaction is started from a coherent vibrational wavepacket, and it may be clarified whether the initial process starts from bond stretching or bond contraction from the gated FT of the real-time spectrum *via* spectrogram analysis. This success makes our system extremely useful for both the analysis and control not only of photochemical reactions, but also of (thermo)chemical reactions.

## 8 Carrier envelope phase control for studying and applying optical-field-material interactions and applications in the frequency domain

While studying the control of generating ultrashort pulses and interactions of such pulses with material, further development has been made related to the absolute phase. The absolute phase is more properly called the carrier envelope phase (CEP).^90–109)^ This may disclose the direct interaction effect of the laser electric field rather than the effect of the laser intensity (squared absolute value of the laser field) on the materials. In the last several decades while using ultrashort laser pulse generation techniques, extremely intense laser pulses could be generated in the sub-femtosecond, and even in attosecond regime.^90–92)^ Nonlinear processes where the effect of the field amplitude, instead of laser intensity is affecting the material response behavior involve “extreme” nonlinearity. For the generation process of attosecond pulses, utilizing few-cycle pulses of which the duration is only a few femtoseconds is employed. In attosecond pulse generation it is important to control the CEP of the few-cycle pulse. Because of the ultrashort time scale and the ultra-intense laser intensity, instead of the interaction between the light intensity and matter, the interaction between the laser-field and matter becomes a target of research, while opening a new field. Already, CEP has disclosed various interesting effects concerning the material-optical electronic field interaction.^93–109)^

Also, during the last few decades, extending the frequency domain with the help of nonlinear optics of intense femtosecond lasers has been successful. Extending to low frequency, into the THz region, has been achieved by optical rectification in a nonlinear crystal. THz has a wide field of applications, such as medicine and security checks. Extending to high frequency by higher harmonics generation (HHG) is a great way to obtain pulses in the XUV to soft X-ray regions.^90)^ The mechanism of the HHG was discussed in terms of a plasma perspective on strong-field multiphoton ionization.^105)^ These X-ray pulses providing structural dynamics information would become complimentary to the electronic dynamics obtained by ultrafast spectroscopy using visible-NIR and UV pulses. Further applications of femtosecond lasers are also possible from a frequency-domain view, since the mode-locked pulse laser has a broad spectrum with a locked frequency separation among longitudinal modes. Utilizing the characteristics, it can be used for frequency metrology.^96,113,114)^

Because of the high peak power of ultrashort pulses compared to longer pulse with the same pulse energy, various applications have been achieved in both laser engineering, including laser machining,^115)^ and laser medicine, as well as laser surgery.^116)^

## 9 Concluding remarks

We have investigated ultrashort visible-near infrared (NIR) pulse generation and their applications for ultrafast spectroscopy. Femtosecond pulses around 800 nm from a Ti:sapphire laser was used as the pump of an optical parametric amplifier in a non-collinear configuration to obtain a broad gain bandwidth by relaxing the phase-matching condition. This non-collinear optical parametric amplifier (NOPA) provides a gain spectrum as broad as 280 nm (from 500 nm to 780 nm) to support the generation of 4.7-fs nearly FT limited pulses. This NOPA is used as both pump and probe pulse in the ultrahigh time-resolved pump-probe experiment. To fully utilize the broad spectrum of the probe pulse, a multi-channel lock-in amplifier (MLA) composed of 128-channel photodiodes was developed and used to detect a broad spectrum with a high signal-to-noise ratio altogether not repeating measurements at each probe wavelength one by one. The combined system of NOPA laser and MLA has clarified detailed mechanisms of ultrafast processes in various systems. The targets being studied were ultrafast processes in varieties of solution including excited state dynamics in molecules and polymers including vibrational relaxation, internal conversion, and intersystem crossing. Also, it was useful in clarifying the mechanism of chemical reactions in solution, including *trans-cis* isomerization. The ultrafast spectroscopy system is also powerful in solid-state physics including novel exotic materials, as well as biophysics of biological systems including pigments relevant to vision and photosynthesis. Even further extensions of real-time vibrational spectroscopy and transition spectroscopy developed were also considered to further disclose structural changes in the transition states during chemical reactions. The measurement and applications of a carrier envelope phase (CEP) of ultrashort pulse are described. The effects of CEP have become important in even shorter pulse and further developments to attosecond spectroscopy and transition-state spectroscopy were briefly discussed.

## Figures and Tables

**Table 1.** History of the optical parametric amplifier (OPA)

Group name	Country	Year	Spectral range	Pulse width (fs)	Number of optical cycles
Kobayashi	Japan	1997	550–700 nm	14	6.8
			960–1150 nm	18	6.5

Riedle	Germany	1997	470–750 nm	16	7.9

De Silvestri	Italy	1998	500–700 nm	11	5.5

Kobayashi	Japan	1998	550–700 nm	6.1	2.9
			900–1300 nm	8.4	2.9

De Silvestri	Italy	1998	500–800 nm	7.3	3.4

Kobayashi	Japan	1999	500–800 nm	4.7	2.2

Kobayashi	Japan	2001	470–800 nm	3.9	1.8

Kobayashi	Japan	2004	780–1600 nm	4.3	1.3

Kobayashi	Japan	2011	420–820 nm	2.4	1.3

## References

[1] Norrish R.G.W. Porter G. (1949) Chemical reactions produced by very high light intensities. Nature 164, 658.18143357

[2] Maiman T.H. (1960) Stimulated optical radiation in ruby. Nature 187, 493–494.

[3] McClung F.J. Hellwarth R.W. (1962) Giant optical pulsations from ruby. J. Appl. Phys. 33, 828–829.

[4] Stalder M. Chai B.H.T. Bass M. (1991) The flashlamp pumped Cr:LiSrAlF_6_ laser. Appl. Phys. Lett. 58, 216–218.

[5] Polanyi J.C. Zewail A.H. (1995) Direct observation of the transition state. Acc. Chem. Res. 28, 119–132.

[6] Kobayashi T. Saito T. Ohtani H. Yabushita A. Saito T. Ohtani H. (2001) Real-time spectroscopy of transition states in bacteriorhodopsin during retinal isomerization. Nature 414, 531–534. 11734850 10.1038/35107042

[7] Pellegrino F. (1983) Ultrafast energy transfer processes in photosynthetic systems probed by picosecond fluorescence spectroscopy. Opt. Eng. 22, 225508.

[8] Boyer A. Déry M. Selles P. Arbour C. Boucher F. (1995) Colour discrimination by forward and reverse photocurrents in bacteriorhodopsin-based photosensor. Biosens. Bioelectron. 10, 415–422.

[9] Hellingwerf K.J. Hendriks J. Gensch T. (2002) On the configurational and conformational changes in photoactive yellow protein that leads to signal generation in ectothiorhodospira halophila. J. Biol. Phys. 28, 395–412. 23345784 10.1023/A:1020360505111 PMC3456738

[10] Imamoto Y. Kataoka M. (2007) Structure and photoreaction of photoactive yellow protein, a structural prototype of the PAS domain superfamily. Photochem. Photobiol. 83, 40–49. 16939366 10.1562/2006-02-28-IR-827

[11] Kuhnert L. (1986) A new optical photochemical memory device in a light-sensitive chemical active medium. Nature 319, 393–394.

[12] Ikeda T. Horiuchi S. Karanjit D.B. Kurihara S. Tazuke S. (1988) Photochemical image storage in polymer liquid crystals. Chem. Lett. 17, 1679–1682.

[13] Korolev N.E. Mokienko I.Y. Poletimov A.E. Shcheulin A.S. (1991) Optical storage material based on doped fluoride crystals. Phys. Status Solidi 127, 327–333.

[14] De Waele V. Schmidhammer U. Mrozek T. Daub J. Riedle E. (2002) Ultrafast bidirectional dihydroazulene/vinylheptafulvene (DHA/VHF) molecular switches: Photochemical ring closure of vinylheptafulvene proven by a two-pulse experiment. J. Am. Chem. Soc. 124, 2438–2439. 11890783 10.1021/ja017132s

[15] Hulin D. Mysyrowicz A. Antonetti A. Migus A. Masselink W.T. Morkoç H. (1986) Ultrafast all-optical gate with subpicosecond ON and OFF response time. Appl. Phys. Lett. 49, 749–751.

[16] Eichmann G. Li Y. Alfano R.R. (1986) Optical binary coded ternary arithmetic and logic. Appl. Opt. 25, 3113–3121. 18235585 10.1364/ao.25.003113

[17] Moulton P. (1982) Ti-doped sapphire: Tunable solid-state laser. Opt. News. 8, 9.

[18] Shih Y. (2003) Quantum imaging, quantum lithography and the uncertainty principle. Eur. Phys. J. D 22, 485–493.

[19] Edamatsu K. Shimizu R. Itoh T. (2002) Measurement of the photonic de broglie wavelength of entangled photon pairs generated by spontaneous parametric down-conversion. Phys. Rev. Lett. 89, 213601. 12443411 10.1103/PhysRevLett.89.213601

[20] Lo H.-P. Yabushita A. Hsu L.-Y. (2014) Exploring steering effects using Bell tests. Phys. Rev. A 89, 022115.

[21] Yabushita A. Kobayashi T. (2004) Spectroscopy by frequency-entangled photon pairs. Phys. Rev. A 69, 3–6.

[22] Lo H.-P. Yabushita A. Luo C.-W. Chen P. Kobayashi T. (2011) Beamlike photon pairs entangled by a 2 × 2 fiber. Phys. Rev. A 84, 022301.

[23] Greenfield S.R. Wasielewski M.R. (1995) Near-transform-limited visible and near-IR femtosecond pulses from optical parametric amplification using Type II β-barium borate. Opt. Lett. 20, 1394–1396. 19862026 10.1364/ol.20.001394

[24] Danielius R. Banfi G.P. Di Trapani P. Righini R. Piskarskas A. Stabinis A. (1993) Traveling-wave parametric generation of widely tunable, highly coherent femtosecond light pulses. J. Opt. Soc. Am. B 10, 2222–2232.

[25] Chuangtan C. Bochang W. Aidong J. Guiming Y. (1985) A new-type ultraviolet SHG crystal—β-BaB2O4. Sci. China Ser. B Chem. Biol. Agric. Med. Earth Sci. 28, 235–243.

[26] Chen, C., Fan, Y.X., Eckardt, R.C. and Byer, R.L. (1987) Recent developments in barium borate. *In* Laser and Nonlinear Optical Materials (Proc SPIE Vol. 0681) (ed. Larry, G.D.). SPIE, Bellingham, Washington, pp. 12–19.

[27] Nisoli M. Stagira S. De Silvestri S. Svelto O. Valiulis G. Varanavicius A. (1998) Parametric generation of high-energy 14.5-fs light pulses at 1.5 µm. Opt. Lett. 23, 630–632. 18084599 10.1364/ol.23.000630

[28] Eimerl D. Davis L. Velsko S. Graham E.K. Zalkin A. (1987) Optical, mechanical, and thermal properties of barium borate. J. Appl. Phys. 62, 1968–1983.

[29] Cerullo G. Nisoli M. Stagira S. De Silvestri S. (1998) Sub-8-fs pulses from an ultrabroadband optical parametric amplifier in the visible. Opt. Lett. 23, 1283–1285. 18087499 10.1364/ol.23.001283

[30] Kobayashi T. Shirakawa A. (2000) Tunable visible and near-infrared pulse generator in a 5 fs regime. Appl. Phys. B 70, S239–S246.

[31] Lee C.-K. Zhang J.-Y. Huang J. Pan C.-L. (2003) Generation of femtosecond laser pulses tunable from 380 nm to 465 nm via cascaded nonlinear optical mixing in a noncollinear optical parametric amplifier with a type-I phase matched BBO crystal. Opt. Express 11, 1702. 19466049 10.1364/oe.11.001702

[32] Di Trapani P. Andreoni A. Banfi G.P. Solcia C. Danielius R. Piskarskas A. (1995) Group-velocity self-matching of femtosecond pulses in noncollinear parametric generation. Phys. Rev. A 51, 3164–3168. 9911955 10.1103/physreva.51.3164

[33] Krylov V. Kalintsev A. Rebane A. Erni D. Wild U.P. (1995) Noncollinear parametric generation in LiIO_3_ and β-barium borate by frequency-doubled femtosecond Ti:sapphire laser pulses. Opt. Lett. 20, 151–153. 19859117 10.1364/ol.20.000151

[34] Sosnowski T.S. Stephens P.B. Norris T.B. (1996) Production of 30-fs pulses tunable throughout the visible spectral region by a new technique in optical parametric amplification. Opt. Lett. 21, 140–142. 19865331 10.1364/ol.21.000140

[35] Danielius R. Di Trapani P. Solcia C. Foggi P. Andreoni A. Piskarskas A. (1996) Matching of group velocities by spatial walk-off in collinear three-wave interaction with tilted pulses. Opt. Lett. 21, 973–975. 19876222 10.1364/ol.21.000973

[36] Wang J. Dunn M.H. Rae C.F. (1997) Polychromatic optical parametric generation by simultaneous phase matching over a large spectral bandwidth. Opt. Lett. 22, 763–765. 18185654 10.1364/ol.22.000763

[37] Wilhelm T. Piel J. Riedle E. (1997) Sub-20-fs pulses tunable across the visible from a blue-pumped single-pass noncollinear parametric converter. Opt. Lett. 22, 1494–1496. 18188279 10.1364/ol.22.001494

[38] Giallorenzi T.G. Tang C.L. (1968) Quantum theory of spontaneous parametric scattering of intense light. Phys. Rev. 166, 225–233.

[39] Kleinman D.A. (1968) Theory of optical parametric noise. Phys. Rev. 174, 1027–1041.

[40] Rabin, H., Tang, C.L. and Bloembergen, N. (1975) Quantum Electronics: A Treatise. Academic Press, New York.

[41] Byer, R.L. and Herbst, R.L. (1977) Parametric oscillation and mixing. *In* Nonlinear Infrared Generation (ed. Shen, Y.R.). Topics in Applied Physics, vol. 16, Springer, Berlin, Heidelberg, pp. 81–137.

[42] Di Trapani P. Andreoni A. Solcia C. Foggi P. Danielius R. Dubietis A. (1995) Matching of group velocities in three-wave parametric interaction with femtosecond pulses and application to traveling-wave generators. J. Opt. Soc. Am. B 12, 2237–2244.

[43] Bor Z. Rácz B. (1985) Group velocity dispersion in prisms and its application to pulse compression and travelling-wave excitation. Opt. Commun. 54, 165–170.

[44] Martinez O.E. (1986) Pulse distortions in tilted pulse schemes for ultrashort pulses. Opt. Commun. 59, 229–232.

[45] Zhang R. Pang D. Sun J. Wang Q. Zhang S. Wen G. (1999) Analytical expressions of group-delay dispersion and cubic phase for four-prism sequence used at other than Brewster’s angle. Opt. Laser Technol. 31, 373–379.

[46] Danielius R. Piskarskas A. Di Trapani P. Andreoni A. Solcia C. Foggi P. (1998) A collinearly phase-matched parametric generator/amplifier of visible femtosecond pulses. IEEE J. Quantum Electron. 34, 459–464.

[47] Ishii N. Tokunaga E. Adachi S. Kimura T. Matsuda H. Kobayashi T. (2004) Optical frequency- and vibrational time-resolved two-dimensional spectroscopy by real-time impulsive resonant coherent Raman scattering in polydiacetylene. Phys. Rev. A 70, 023811.

[48] Nishimura K. Tokunaga E. Kobayashi T. (2004) Sub-5-fs two-dimensional spectroscopy of pseudoisocyanine J-aggregates. Chem. Phys. Lett. 395, 114–119.

[49] Ikuta M. Yuasa Y. Kimura T. Matsuda H. Kobayashi T. (2004) Phase analysis of vibrational wave packets in the ground and excited states in polydiacetylene. Phys. Rev. B 70, 214301.

[50] Yuasa Y. Ikuta M. Kobayashi T. Kimura T. Matsuda H. (2005) Vibrational chirp in the dynamic Stokes-shift process due to ultrafast geometrical relaxation in a polydiacetylene. Phys. Rev. B 72, 134302.

[51] Wang Z. Otsubo T. Kobayashi T. (2006) Chirped modulation of molecular vibration in quinoidal thiophene after sub-5 fs excitation. Chem. Phys. Lett. 430, 45–50. 10.1063/1.2213977 16942130

[52] Kobayashi T. Yabushita A. Saito T. Ohtani H. Tsuda M. (2007) Sub-5-fs real-time spectroscopy of *transition states* in bacteriorhodopsin during retinal isomerization. Photochem. Photobiol. 83, 363–369. 17132067 10.1562/2006-08-19-IR-1006

[53] Iwakura I. Yabushita A. Kobayashi T. (2007) Sum and difference frequency mixing of molecular vibrations in a polymer under high-density optical excitation. Phys. Rev. B 76, 052201.

[54] Colonna A. Yabushita A. Iwakura I. Kobayashi T. (2007) Chirped molecular vibration in a stilbene derivative in solution. Chem. Phys. 341, 336–343.

[55] Du J. Wang Z. Feng W. Yoshino K. Kobayashi T. (2008) Simultaneous measurement of electronic and vibrational dynamics to clarify a geometrical relaxation process in a conjugated polymer. Phys. Rev. B 77, 195205.

[56] Zhang J. Wang Z. Kobayashi T. (2008) Vibrational fine structures revealed by the real-time vibrational phase and amplitude in MEH-PPV using few cycle pulses. Phys. Rev. B 77, 153202.

[57] Kobayashi T. Iwakura I. Yabushita A. (2008) Excitonic and vibrational nonlinear processes in a polydiacetylene studied by a few-cycle pulse laser. New J. Phys. 10, 065016.

[58] Kobayashi T. Wang Y. Wang Z. Iwakura I. (2008) Circa conservation of vibrational energy among three strongly coupled modes of a cyanine dye molecule studied by quantum-beat spectroscopy with a 7 fs laser. Chem. Phys. Lett. 466, 50–55.

[59] Teramoto T. Wang Z. Kobryanskii V.M. Taneichi T. Kobayashi T. (2009) Ultrafast real-time vibronic coupling of a breather soliton in trans-polyacetylene using a laser pulse with few cycles. Phys. Rev. B 79, 033202.

[60] Yabushita A. Kobayashi T. (2009) Primary conformation change in bacteriorhodopsin on photoexcitation. Biophys. J. 96, 1447–1461. 19217861 10.1016/j.bpj.2008.10.050 PMC2717252

[61] Sugita A. Saito T. Kano H. Yamashita M. Kobayashi T. (2001) Wave packet dynamics in a quasi-one-dimensional metal-halogen complex studied by ultrafast time-resolved spectroscopy. Phys. Rev. Lett. 86, 2158–2161. 11289879 10.1103/PhysRevLett.86.2158

[62] Ozawa A. Takimiya K. Otsubo T. Kobayashi T. (2005) Sub-5 fs time-resolved dynamic Franck-Condon overlaps associated with the S1 → S0 stimulated transition in oligothiophene 13-mer. Chem. Phys. Lett. 409, 224–229.

[63] Kobayashi T. Wang H. Wang Z. Otsubo T. (2006) Confined breather-type excitation in a quinoidal thiophene after sub-5 fs pulse excitation. J. Chem. Phys. 125, 044103. 10.1063/1.2213977 16942130

[64] Yabushita A. Kobayashi T. (2010) Ultrafast spectroscopy of oxyhemoglobin during photodissociation. J. Phys. Chem. B 114, 11654–11658. 20712382 10.1021/jp103593q

[65] Yabushita A. Lee Y.-H. Kobayashi T. (2010) Development of a multiplex fast-scan system for ultrafast time-resolved spectroscopy. Rev. Sci. Instrum. 81, 063110. 20590228 10.1063/1.3455809

[66] Hsu C.-C. Wang Y.-T. Yabushita A. Luo C.-W. Hsiao Y.-N. Lin S.-H. (2011) Environment-dependent ultrafast photoisomerization dynamics in azo dye. J. Phys. Chem. A 115, 11508–11514. 21916471 10.1021/jp2051307

[67] Du J. Nakata K. Jiang Y. Tokunaga E. Kobayashi T. (2011) Spectral modulation observed in Chl-*a* by ultrafast laser spectroscopy. Opt. Express 19, 22480–22485. 22109125 10.1364/OE.19.022480

[68] Yabushita A. Kobayashi T. Tsuda M. (2012) Time-resolved spectroscopy of ultrafast photoisomerization of octopus rhodopsin under photoexcitation. J. Phys. Chem. B 116, 1920–1926. 22251430 10.1021/jp209356s

[69] Iwakura I. Kaneko Y. Hayashi S. Yabushita A. Kobayashi T. (2013) The reaction mechanism of claisen rearrangement obtained by transition state spectroscopy and single direct-dynamics trajectory. Molecules 18, 1995–2004. 23381025 10.3390/molecules18021995 PMC6270580

[70] Kobayashi T. (2013) Development of ultrafast spectroscopy and reaction mechanisms studied by the observation of ultrashort-life species and transition states. Bull. Chem. Soc. Jpn. 86, 167–182.

[71] Kobayashi T. Iiyama T. Okamura K. Du J. Masuda T. (2013) Ultrafast electronic relaxation and vibrational dynamics in a polyacetylene derivative. Chem. Phys. Lett. 567, 6–13.

[72] Kobayashi T. Nie Z. Du J. Okamura K. Kataura H. Sakakibara Y. (2013) Electronic relaxation and coherent phonon dynamics in semiconducting single-walled carbon nanotubes with several chiralities. Phys. Rev. B 88, 035424.

[73] Iwakura I. Yabushita A. Liu J. Okamura K. Kezuka S. Kobayashi T. (2013) A new reaction mechanism of Claisen rearrangement induced by few-optical-cycle pulses: Demonstration of nonthermal chemistry by femtosecond vibrational spectroscopy. Pure Appl. Chem. 85, 1991–2004.

[74] Kobayashi T. Nie Z. Xue B. Kataura H. Sakakibara Y. Miyata Y. (2014) Real-time spectroscopy of single-walled carbon nanotubes for negative time delays by using a few-cycle pulse laser. J. Phys. Chem. C 118, 3285–3294.

[75] Yabushita A. Juang D.-Y. Kao C.-H. Baltuška A. Kobayashi T. (2014) Generation of multi-color carrier-envelope phase locked pulse with continuous color tunability. Opt. Commun. 315, 310–316.

[76] Nakata K. Tokunaga E. Du J. Xue B. Miyazaki J. Seto K. (2014) Sub-10 fs spectroscopy of K-TCNQ crystal for observation of intramolecular vibration modulation in melting of the Peierls dimer. Phys. Rev. B 90, 085119.

[77] Kobayashi, T. (2016) Ultrafast spectroscopy of coherent phonon in carbon nanotubes using sub-5-fs visible pulses. *In* AIP Conference Proceedings, Vol. 1709. AIP, 020001.

[78] Xue B. Yabushita A. Kobayashi T. (2016) Ultrafast dynamics of uracil and thymine studied using a sub-10 fs deep ultraviolet laser. Phys. Chem. Chem. Phys. 18, 17044–17053. 27299165 10.1039/c5cp07861j

[79] Kida Y. Liu J. Kobayashi T. (2011) Single 10-fs deep-ultraviolet pulses generated by broadband four-wave mixing and high-order dispersion compensation. Appl. Phys. B 105, 675–679.

[80] Du J. Harra J. Virkki M. Mäkelä J.M. Leng Y. Kauranen M. (2016) Surface-enhanced impulsive coherent vibrational spectroscopy. Sci. Rep. 6, 36471. 27812020 10.1038/srep36471 PMC5095601

[81] Hashimoto S. Yabushita A. Kobayashi T. Okamura K. Iwakura I. (2018) Direct observation of the change in transient molecular structure of 9,9′-bianthryl using a 10 fs pulse UV laser. Chem. Phys. 512, 128–134.

[82] Hung C.-C. Yabushita A. Kobayashi T. Chen P.-F. Liang K.S. (2017) Ultrafast relaxation dynamics of nitric oxide synthase studied by visible broadband transient absorption spectroscopy. Chem. Phys. Lett. 683, 619–624.

[83] Du J. Yuan W. Xing X. Miyatake T. Tamiaki H. Kobayashi T. (2017) Spectral modulation observed in artificial photosynthetic complexes by real-time vibrational spectroscopy. Chem. Phys. Lett. 683, 154–159.

[84] Hung C.-C. Chen X.-R. Ko Y.-K. Kobayashi T. Yang C.-S. Yabushita A. (2017) Schiff base proton acceptor assists photoisomerization of retinal chromophores in bacteriorhodopsin. Biophys. J. 112, 2503–2519. 28636908 10.1016/j.bpj.2017.05.015 PMC5479150

[85] Yamakita Y. Yokoyama N. Xue B. Shiokawa N. Harabuchi Y. Maeda S. (2019) Femtosecond electronic relaxation and real-time vibrational dynamics in 2′-hydroxychalcone. Phys. Chem. Chem. Phys. 21, 5344–5358. 30484793 10.1039/c8cp06405a

[86] Jarota A. Pastorczak E. Tawfik W. Xue B. Kania R. Abramczyk H. (2019) Exploring the ultrafast dynamics of a diarylethene derivative using sub-10 fs laser pulses. Phys. Chem. Chem. Phys. 21, 192–204. 10.1039/c8cp05882b 30516769

[87] Hashimoto S. Hamada K. Iwakura I. Yabushita A. Kobayashi T. Fujita H. (2019) Photochemical reaction mechanisms of 4,5-dimethoxy-2-nitrobenzyl acetate analysed by a sub-10 fs near-ultraviolet pulse laser. Chem. Phys. 524, 70–76.

[88] Tu C.-M. Yeh T.-T. Tzeng W.-Y. Chen Y.-R. Chen H.-J. Ku S.-A. (2015) Manifestation of a second Dirac surface state and bulk bands in THz radiation from topological insulators. Sci. Rep. 5, 14128. 26370337 10.1038/srep14128 PMC4569898

[89] Polli D. Lüer L. Cerullo G. (2007) High-time-resolution pump-probe system with broadband detection for the study of time-domain vibrational dynamics. Rev. Sci. Instrum. 78, 103108. 17979407 10.1063/1.2800778

[90] Drescher M. Hentschel M. Kienberger R. Tempea G. Spielmann C. Reider G.A. (2001) X-ray pulses approaching the attosecond frontier. Science 291, 1923–1927. 11239146 10.1126/science.1058561

[91] Paul P.M. Toma E.S. Breger P. Mullot G. Augé F. Balcou P. (2001) Observation of a train of attosecond pulses from high harmonic generation. Science 292, 1689–1692. 11387467 10.1126/science.1059413

[92] Baltuška A. Udem T. Uiberacker M. Hentschel M. Goulielmakis E. Gohle C. (2003) Attosecond control of electronic processes by intense light fields. Nature 421, 611–615. 12571590 10.1038/nature01414

[93] Dietrich P. Krausz F. Corkum P.B. (2000) Determining the absolute carrier phase of a few-cycle laser pulse. Opt. Lett. 25, 16–18. 18059767 10.1364/ol.25.000016

[94] Xu L. Hänsch T.W. Spielmann C. Poppe A. Brabec T. Krausz F. (1996) Route to phase control of ultrashort light pulses. Opt. Lett. 21, 2008–2010. 19881875 10.1364/ol.21.002008

[95] Jones D.J. Diddams M. Ranka R. Stentz G. Windeler C. Hall G.A. (2000) Carrier-envelope phase control of femtosecond mode-locked lasers and direct optical frequency synthesis. Science 288, 635–639. 10784441 10.1126/science.288.5466.635

[96] Diddams S.A. Jones D.J. Ye J. Cundiff S.T. Hall J.L. Ranka J.K. (2000) Direct link between microwave and optical frequencies with a 300 THz femtosecond laser comb. Phys. Rev. Lett. 84, 5102–5105. 10990877 10.1103/PhysRevLett.84.5102

[97] Kakehata M. Takada H. Kobayashi Y. Torizuka K. Fujihira Y. Homma T. (2001) Single-shot measurement of carrier-envelope phase changes by spectral interferometry. Opt. Lett. 26, 1436–1438. 18049630 10.1364/ol.26.001436

[98] Kobayashi Y. Torizuka K. (2000) Measurement of the optical phase relation among subharmonic pulses in a femtosecond optical parametric oscillator. Opt. Lett. 25, 856–858. 18064207 10.1364/ol.25.000856

[99] Mehendale M. Mitchell S.A. Likforman J.-P. Villeneuve D.M. Corkum P.B. (2000) Method for single-shot measurement of the carrier envelope phase of a few-cycle laser pulse. Opt. Lett. 25, 1672–1674. 18066311 10.1364/ol.25.001672

[100] Telle H.R. Steinmeyer G. Dunlop A.E. Stenger J. Sutter D.H. Keller U. (1999) Carrier-envelope offset phase control: A novel concept for absolute optical frequency measurement and ultrashort pulse generation. Appl. Phys. B 69, 327–332.

[101] Zhou J. Christov I.P. Taft G. Huang C.-P. Murnane M.M. Kapteyn H.C. (1994) Pulse evolution in a broad-bandwidth Ti:sapphire laser. Opt. Lett. 19, 1149–1151. 19844559 10.1364/ol.19.001149

[102] de Bohan A. Piraux B. Ponce L. Taïeb R. Véniard V. Maquet A. (2002) Direct and indirect pathways in strong field atomic ionization dynamics. Phys. Rev. Lett. 89, 113002. 12225139 10.1103/PhysRevLett.89.113002

[103] Paulus G.G. Grasbon F. Walther H. Villoresi P. Nisoli M. Stagira S. (2001) Absolute-phase phenomena in photoionization with few-cycle laser pulses. Nature 414, 182–184. 11700551 10.1038/35102520

[104] Yabushita A. Juang D.-Y. Kao C.-H. Baltuška A. Kobayashi T. (2014) Generation of multi-color carrier-envelope phase locked pulse with continuous color tunability. Opt. Commun. 315, 310–316.

[105] Corkum P.B. (1993) Plasma perspective on strong field multiphoton ionization. Phys. Rev. Lett. 71, 1994–1997. 10054556 10.1103/PhysRevLett.71.1994

[106] Baltuška A. Fuji T. Kobayashi T. (2002) Controlling the carrier-envelope phase of ultrashort light pulses with optical parametric amplifiers. Phys. Rev. Lett. 88, 133901. 11955097 10.1103/PhysRevLett.88.133901

[107] Malitson I.H. (1963) A redetermination of some optical properties of calcium fluoride. Appl. Opt. 2, 1103–1107.

[108] Baltuška A. Kobayashi T. (2002) Adaptive shaping of two-cycle visible pulses using a flexible mirror. Appl. Phys. B 75, 427–443.

[109] Cerullo G. Baltuška A. Mücke O.D. Vozzi C. (2011) Few-optical-cycle light pulses with passive carrier-envelope phase stabilization. Laser Photonics Rev. 5, 323–351.

[110] Kobayashi T. Wang Z. (2008) Spectral oscillation in optical frequency-resolved quantum-beat spectroscopy with a few-cycle pulse laser. IEEE J. Quantum Electron. 44, 1232–1241.

[111] Kobayashi T. Wang Z. (2008) Correlations of instantaneous transition energy and intensity of absorption peaks during molecular vibration: Toward potential hyper-surface. New J. Phys. 10, 065015.

[112] Hashimoto S. Hamada K. Iwakura I. Yabushita A. Kobayashi T. Fujita H. (2019) Photochemical reaction mechanisms of 4,5-dimethoxy-2-nitrobenzyl acetate analysed by a sub-10 fs near-ultraviolet pulse laser. Chem. Phys. 524, 70–76.

[113] Asahara A. Minoshima K. (2019) Coherent multi-comb pulse control demonstrated in polarization-modulated dual-comb spectroscopy technique. Appl. Phys. Express 12, 072014.

[114] Nakajima Y. Hata Y. Minoshima K. (2019) All-polarization-maintaining, polarization-multiplexed, dual-comb fiber laser with a nonlinear amplifying loop mirror. Opt. Express 27, 14648–14656. 31163909 10.1364/OE.27.014648

[115] Phillips K.C. Gandhi H.H. Mazur E. Sundaram S.K. (2015) Ultrafast laser processing of materials: A review. Adv. Opt. Photonics 7, 684–712.

[116] Nagy Z.Z. McAlinden C. (2015) Femtosecond laser cataract surgery. Eye and Vision 2, 11. 26605364 10.1186/s40662-015-0021-7 PMC4655462

